# The centromeric histone CenH3 is recruited into the tombusvirus replication organelles

**DOI:** 10.1371/journal.ppat.1010653

**Published:** 2022-06-29

**Authors:** Paulina Alatriste Gonzalez, Peter D. Nagy

**Affiliations:** Department of Plant Pathology, University of Kentucky, Lexington, Kentucky, United States of America; Agriculture and Agri-Food Canada, CANADA

## Abstract

Tombusviruses, similar to other (+)RNA viruses, exploit the host cells by co-opting numerous host components and rewiring cellular pathways to build extensive virus-induced replication organelles (VROs) in the cytosol of the infected cells. Most molecular resources are suboptimal in susceptible cells and therefore, tomato bushy stunt virus (TBSV) drives intensive remodeling and subversion of many cellular processes. The authors discovered that the nuclear centromeric CenH3 histone variant (Cse4p in yeast, CENP-A in humans) plays a major role in tombusvirus replication in plants and in the yeast model host. We find that over-expression of CenH3 greatly interferes with tombusvirus replication, whereas mutation or knockdown of CenH3 enhances TBSV replication in yeast and plants. CenH3 binds to the viral RNA and acts as an RNA chaperone. Although these data support a restriction role of CenH3 in tombusvirus replication, we demonstrate that by partially sequestering CenH3 into VROs, TBSV indirectly alters selective gene expression of the host, leading to more abundant protein pool. This in turn helps TBSV to subvert pro-viral host factors into replication. We show this through the example of hypoxia factors, glycolytic and fermentation enzymes, which are exploited more efficiently by tombusviruses to produce abundant ATP locally within the VROs in infected cells. Altogether, we propose that subversion of CenH3/Cse4p from the nucleus into cytosolic VROs facilitates transcriptional changes in the cells, which ultimately leads to more efficient ATP generation *in situ* within VROs by the co-opted glycolytic enzymes to support the energy requirement of virus replication. In summary, CenH3 plays both pro-viral and restriction functions during tombusvirus replication. This is a surprising novel role for a nuclear histone variant in cytosolic RNA virus replication.

## Author summary

Biogenesis of viral replication organelles (VROs) containing membrane-bound viral replicase complexes is critical for replication of (+)RNA viruses. To achieve efficient VRO formation, RNA viruses reprogram host gene transcription through incompletely characterized processes. Using tombusviruses and the model host yeast, the authors discovered that the centromeric Histone 3 variant (CenH3 or CENP-A) is recruited into tombusvirus VROs. Tombusvirus replication protein and the viral RNA interact with CenH3 leading to partial sequestration of CenH3 into cytosolic VROs from the nucleus. The subversion of CenH3 leads to selective re-programming of gene expression of the host, resulting in increased production of glycolytic and fermentation enzymes. These enzymes are exploited by tombusviruses to produce ATP locally within the VROs to achieve robust replication.

## Introduction

Positive-strand (+)RNA viruses use the abundant resources of the host cells to build large viral replication compartments/organelles (VROs), which support their replication in a membranous protective microenvironment [1–9]. Tomato bushy stunt virus (TBSV), a (+)RNA virus, is intensively studied to decipher virus-host interactions, virus replication and recombination. An emerging theme from TBSV studies is that this cytosolic replicating virus dramatically remodels subcellular membranes, hijacks various transport vesicles and co-opts numerous host proteins to facilitate various steps in the robust viral replication process [10,11]. Interestingly, however, the originally available resources in the uninfected host cells provide suboptimal conditions for robust TBSV replication. Accordingly, ever-increasing data show that TBSV rewires metabolic processes, alters the lipid compositions of the targeted endomembranes and organelles, and induces host gene expression to increase the abundance of host factors, which are co-opted for TBSV replication in the infected cells [10–12].

TBSV codes for two viral replication proteins, termed p33 and p92^pol^, which are essential for virus replication [13,14]. These replication proteins induce all the above cellular changes with major assistance from co-opted host enzymes and pathways [11,15,16]. Therefore, a major frontier in virus-host interaction studies is to advance our understanding how a (+)RNA virus can rewire cellular pathways and optimize the cellular milieu that then will support robust viral RNA replication. Yet, the picture of virus-host interactions is further complicated by host responses, including an arsenal of restriction factors, which inhibit the viral replication.

Using a library of temperature-sensitive mutants of yeast (a model host for TBSV), we identified Cse4 centromeric H3 protein variant as a restriction factor for TBSV replication [17]. Based on a protein network analysis [18], we found that Cse4 is one of the most highly connected nodes among the ~500 host factors identified, which affect TBSV replication, recombination or TBSV-host interaction in yeast [19–24]. This is a surprising discovery, because the DNA-binding nuclear histone proteins are not known to function as antiviral proteins against the cytosolic RNA viruses. Therefore, we decided to dissect the function of Cse4 and the plant CenH3 in TBSV replication.

The nucleus of a eukaryotic cell is full of nucleic acid binding proteins, which can potentially be used by the host to fight viral infections. Indeed, many well-characterized nuclear proteins are shuttling in and out of the nucleus, making possible that these cellular proteins could also function in the cytosol [16,25–28]. The histone H3 variant, CenH3 is essential for chromosome segregation by marking the centromere. This protein is so conserved in eukaryotes that the yeast Cse4p can complement the human CENP-A [29]. Nucleosomes containing CenH3 bind to long noncoding RNAs (called cenRNA) in the nucleus, which helps CenH3 to be localized to the centromeric portion of chromosomes [30,31]. However, CENP-A also localizes to noncentromeric regions (130 sites in yeast and 11,000 ectopic locations in the human chromosomes) in S phase [32,33]. These noncentromeric sites frequently represent intergenic and promoter regions with high histone turnover and transcriptional hotspots [33–36]. In yeast, high levels of Cse4p in promoter regions were related to down-regulation of gene expression [35]. Moreover, mislocalization and overexpression of CenH3 has been found in many cancers and associated with aneuploidy in Drosophila [37–40].

In this paper, we studied the role of CenH3 in TBSV replication in yeast, plants and *in vitro*. Based on knockdown, mutation or over-expression experiments, we showed that CenH3/Cse4p acts as a cellular restriction factor against TBSV replication. CenH3/Cse4p was found to be partially re-targeted from the nucleus into the cytosolic VROs. *In vitro* works showed that CenH3/Cse4p binds to the viral RNA and acts as an RNA chaperone. Co-purification and pulldown experiments demonstrated interaction between CenH3/Cse4p and the viral p33 replication protein. However, subsequent analysis showed that TBSV hijacks CenH3/Cse4p into VROs to sequester this histone 3 variant away from the nucleus, which affects the expression of a set of host genes. These genes include pro-tombusviral host factors. We chose to further test the role of CenH3/Cse4p in regulating the glycolytic and fermentation pathways, which are co-opted by tombusviruses. These pathways are usurped by TBSV to provide plentiful ATP within VROs to fuel the activities of additional co-opted host proteins, such as Hsp70, the ESCRT-associated Vps4 AAA ATPase and DEAD-box helicases needed for robust viral replication [41–46]. Altogether, CenH3/Cse4p plays both pro-viral and restriction functions during tombusvirus replication.

## Results

### The nuclear CenH3 histone variant restricts tombusvirus replication in yeast and plants

**Fig 1:**
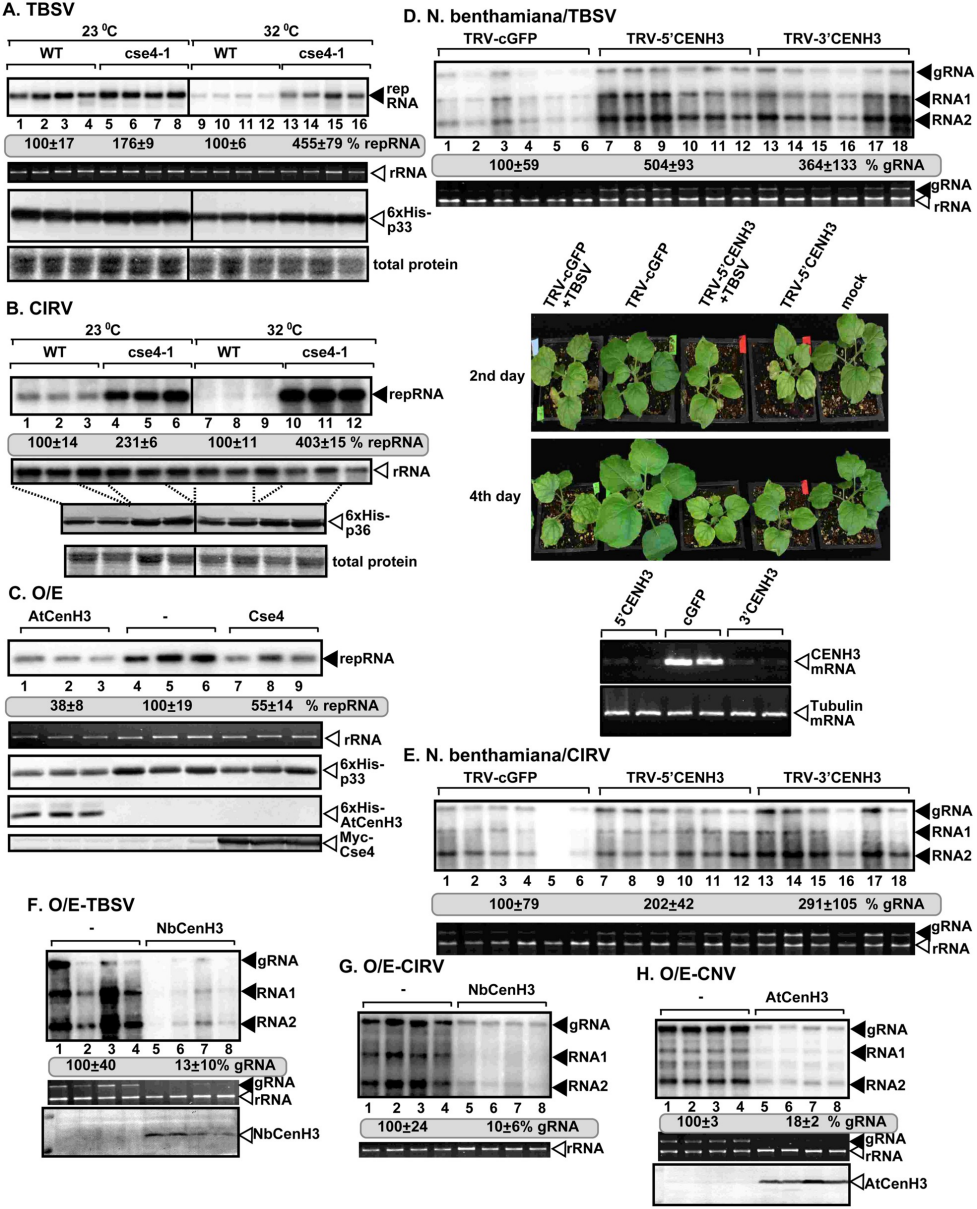
The essential centromeric histone variant CenH3 is a restriction factor of tombusvirus replication in yeast and plants. (A and B) Top: Northern blot analyses show increased TBSV (A) or CIRV (B) repRNA accumulation in WT and cse4-1 yeasts grown at permissive and semi-permissive (32°C) temperatures. Middle: The accumulation level of repRNA was normalized based on 18S rRNA levels. Bottom: The accumulation levels of His_6_-p33 or the CIRV His_6_-p36 replication protein were measured by western blotting with anti-His antibody. (C) The effect of CenH3 overexpression on TBSV repRNA replication in yeast cells. Top: The 3’ end specific probe used for northern blot shows a reduction in the accumulation of TBSV repRNA in yeast expressing *A*. *thaliana* His_6_-tagged CenH3 (lanes 1–3) or *S*. *cerevisiae* Myc-tagged Cse4p (lanes 7–9) in comparison with the empty vector control (lanes 4–6). Bottom: Western blot analyses of the level of His_6_-p33, His_6_-AtCenH3 with anti-His antibody and Myc-Cse4 with anti-Myc antibody. (D) VIGS-based knockdown of CenH3 expression leads to increased level of TBSV replication in *N*. *benthamiana* plants. Top: Northern blot analysis of tombusvirus gRNA and sgRNA accumulation in CenH3-silenced plants inoculated with TBSV. VIGS was performed via agroinfiltration of tobacco rattle virus (TRV) vector carrying 5’ or 3’-terminal NbCenH3 sequences or 3’-terminal GFP sequences as control. Middle: Ethidium bromide-stained gel shows ribosomal RNA levels in each sample as a loading control. Bottom: CenH3-silencing restricts the growth of plants. NbCenH3 mRNA levels were analyzed by semi-quantitative RT-PCR in the silenced and control plants. Tubulin mRNA was used as a control. (E) Accumulation of CIRV gRNA and sgRNA in CenH3-silenced *N*. *benthamiana* plants was measured by Northern blot analysis. See further details in panel D. (F-H) Northern blot analyses of tombusvirus gRNA and sgRNA accumulation in *N*. *benthamiana* plants expressing CenH3 and inoculated with TBSV (F), CIRV (G) or CNV (H). Samples were taken 48 h (F), 72 h (G) or 84 h (H) after virus inoculation. Each experiment was performed at least three times.

To explore the possible role of Cse4p (CenH3) in tombusvirus replication, we used the temperature-sensitive haploid yeast strain with a mutated single copy of *cse4-1* [17,47]. Partial inhibition of Cse4p by growing the yeast cse4-1 strain at the semi-permissive temperature (32°C) resulted in a ~4-fold increased level of TBSV replicon (rep)RNA [48,49] replication when compared with the BY4741 yeast strain carrying the WT copy of *CSE4* (Fig 1A, compare lanes 13–16 to 9–12). TBSV replication was also higher in the cse4-1 strain than in the WT strain even at the permissive temperature (23°C, Fig 1A). This might indicate that a novel activity, not the canonical essential function of Cse4p in chromosome segregation, provides inhibitory effect against TBSV replication. Western blot analysis revealed that the tombusvirus p33 replication protein was expressed close to WT level in the cse4-1 strain (Fig 1A). We also tested the closely-related carnation Italian ringspot virus (CIRV), which replicates on the boundary membranes of mitochondria in contrast with the peroxisome-associated TBSV. CIRV replication is also increased by ~4-fold in cse4-1 strain at the semi-permissive temperature (Fig 1B). These findings suggest that Cse4p is a restriction factor for tombusvirus replication in different subcellular environments.

We used another approach to test the restriction function of CenH3 by expressing the WT *Arabidopsis* CenH3 in yeast replicating TBSV. We observed ~3-fold inhibition of TBSV repRNA accumulation in comparison with the control yeast (Fig 1C). Similarly, over-expression of the yeast Cse4p also inhibited TBSV repRNA replication in yeast (Fig 1C). These experiments confirmed that under these conditions, CenH3/Cse4 acts as a restriction factor during TBSV replication.

To further explore if the plant CenH3 acts as a restriction factor of tombusvirus replication, we used a virus-induced gene silencing (VIGS) approach to deplete CenH3 level in *Nicotiana benthamiana* (Fig 1D, bottom panels) [50]. Replication of TBSV genomic (g)RNA was increased by ~3-to-5-fold in the CenH3 knockdown plants when compared to the non-silenced control plants two days after inoculation (Fig 1D, lanes 7–18 versus 1–6). Knockdown of CenH3 (TRV-5’CENH3, Fig 1D) rendered the plants smaller than the control (TRV-cGFP) plants, yet the knockdown plants supported higher levels of TBSV replication, suggesting that low CenH3 expression makes the plants more suited to support TBSV replication. Comparable experiments with CIRV showed that the CenH3 knockdown plants are indeed highly supportive of tombusvirus replication (Fig 1E).

To further test the restriction function of CenH3 against tombusvirus replication in plants, we transiently expressed either NbCenH3 or AtCenH3 in *N*. *benthamiana* followed by inoculation of the same leaves with two peroxisome-associated tombusviruses (i.e. TBSV and the closely-related cucumber necrosis virus, CNV) and the mitochondrial membrane-associated CIRV. Northern blot analysis revealed ~8-to-10-fold reduction in TBSV, CNV and CIRV gRNA accumulation in the inoculated leaves (Fig 1F–1H). Therefore, all the above data support a strong tombusvirus restriction function of the plant CenH3 histone variant.

### Recruitment of the nuclear CenH3 histone variant into the tombusvirus replication organelles in plants

**Fig 2:**
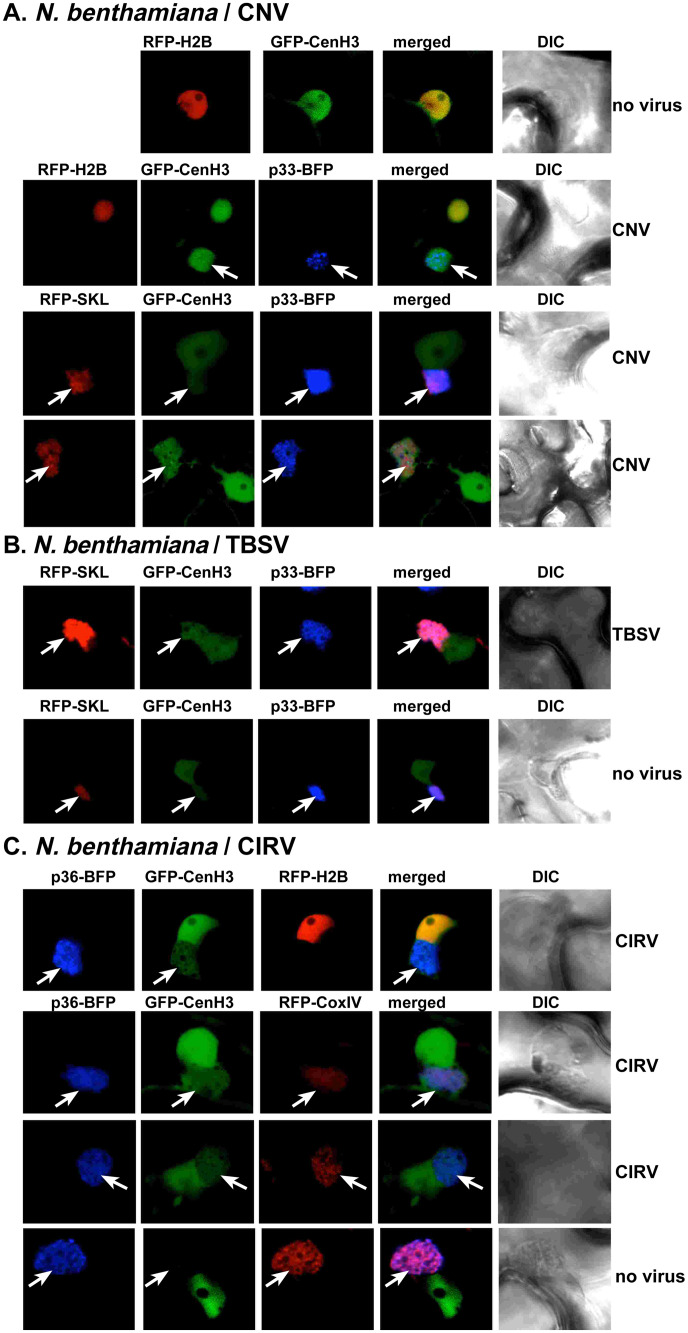
Re-distribution of nuclear CenH3 to the cytosolic sites of viral replication in plants. Confocal laser microscopy images show the localization of GFP-CenH3 in *N*. *benthamiana* cells. (A) First panel: In the absence of virus replication, GFP-CenH3 localizes solely in the nucleus, as shown by its co-localization with the histone RFP-H2B. Second panel: Co-localization of p33-BFP replication protein and GFP-CenH3 in cells replicating CNV. The VROs are marked with white arrows. Third and fourth panels: The re-distributed GFP-CenH3 is present in the VROs, marked by p33-BFP and RFP-SKL peroxisomal marker protein. Note that a portion of GFP-CenH3 remains in the nucleus. (B) GFP-CenH3 partially re-localizes into the VRO-like structures, in the absence of TBSV replication when only TBSV p33-BFP is expressed. (C) First panel: Co-localization of CIRV p36-BFP replication protein and GFP-CenH3 in cells replicating CIRV. Second and third panels: GFP-CenH3 is re-distributed into the CIRV-induced VROs consisting of aggregated mitochondrial membranes, marked with both p36-BFP and RFP-CoxIV. Fourth panel: GFP-CenH3 does not re-localize into the VRO-like structures, in the absence of CIRV replication when only p36-BFP replication protein is expressed.

To test if the restriction function of CenH3 is performed in the nucleus or in the cytosol, where tombusviruses assemble the large viral replication organelles (VROs), we co-expressed TBSV p33-BFP replication protein and the GFP-tagged *Arabidopsis* CenH3, the ortholog of the yeast Cse4, in transgenic *N*. *benthamiana* leaves expressing the RFP-H2B (histone H2B) nuclear marker protein (Fig 2A). Confocal laser microscopy analysis revealed the co-localization of p33-BFP and GFP-CenH3 in *N*. *benthamiana* cells replicating CNV (Fig 2A). Interestingly, a portion of GFP-CenH3 was still localized in the nucleus marked by the RFP-H2B marker protein in plant cells infected with CNV (Fig 2A). In contrast, GFP-CenH3 was exclusively localized to the nucleus in mock-inoculated plant leaves under these transient expression conditions (Fig 2A, top image). Importantly, the re-localized GFP-CenH3 in the cytosol was present in the TBSV VROs marked by both p33-BFP and RFP-SKL peroxisome luminar marker protein (Fig 2B). The expression of p33-BFP alone (in the absence of TBSV infection) facilitated the partial re-localization of GFP-CenH3 into VRO-like structures (Fig 2B), albeit this process was not as robust as in the case of TBSV or CNV infections. We also performed comparable experiments with the mitochondrial CIRV in either transgenic RFP-H2B or WT *N*. *benthamiana* plants. The results showed partial re-localization of GFP-CenH3 into CIRV-induced VRO structures marked by p36-BFP and RFP-CoxIV mitochondrial marker protein (Fig 2C). The expression of p36-BFP alone (in the absence of CIRV infection) did not induce the re-localization of GFP-CenH3 into VRO-like structures (Fig 2C). Based on these results, we suggest that tombusvirus infections of *N*. *benthamiana* plants induce the partial re-localization of the nuclear GFP-CenH3 into the cytosolic VROs.

We also performed subcellular localization experiments in WT yeast cells expressing YFP-Cse4 and co-expressing CFP-p33 together with p92^pol^ and the repRNA to induce VRO formation [51]. Time point experiments revealed the partial co-localization of YFP-Cse4 with CFP-p33 at 12 h time point after induction of protein expression (S1 Fig). The co-localization of YFP-Cse4 and CFP-p33 was even more pronounced at the 16 h and 24 h time points (S1 Fig). This is in contrast with the nuclear localization of YFP-Cse4 in WT yeast in the absence of viral components (S1 Fig). Based on these data, we propose that Cse4p is partially re-localized from the nucleus into the cytosolic VROs marked by p33 replication protein in yeast. Altogether, CenH3 and the orthologous Cse4p are re-targeted by TBSV in plant and yeast cells.

### The CenH3 histone variant is an RNA chaperone inhibiting tombusvirus replication *in vitro*

**Fig 3:**
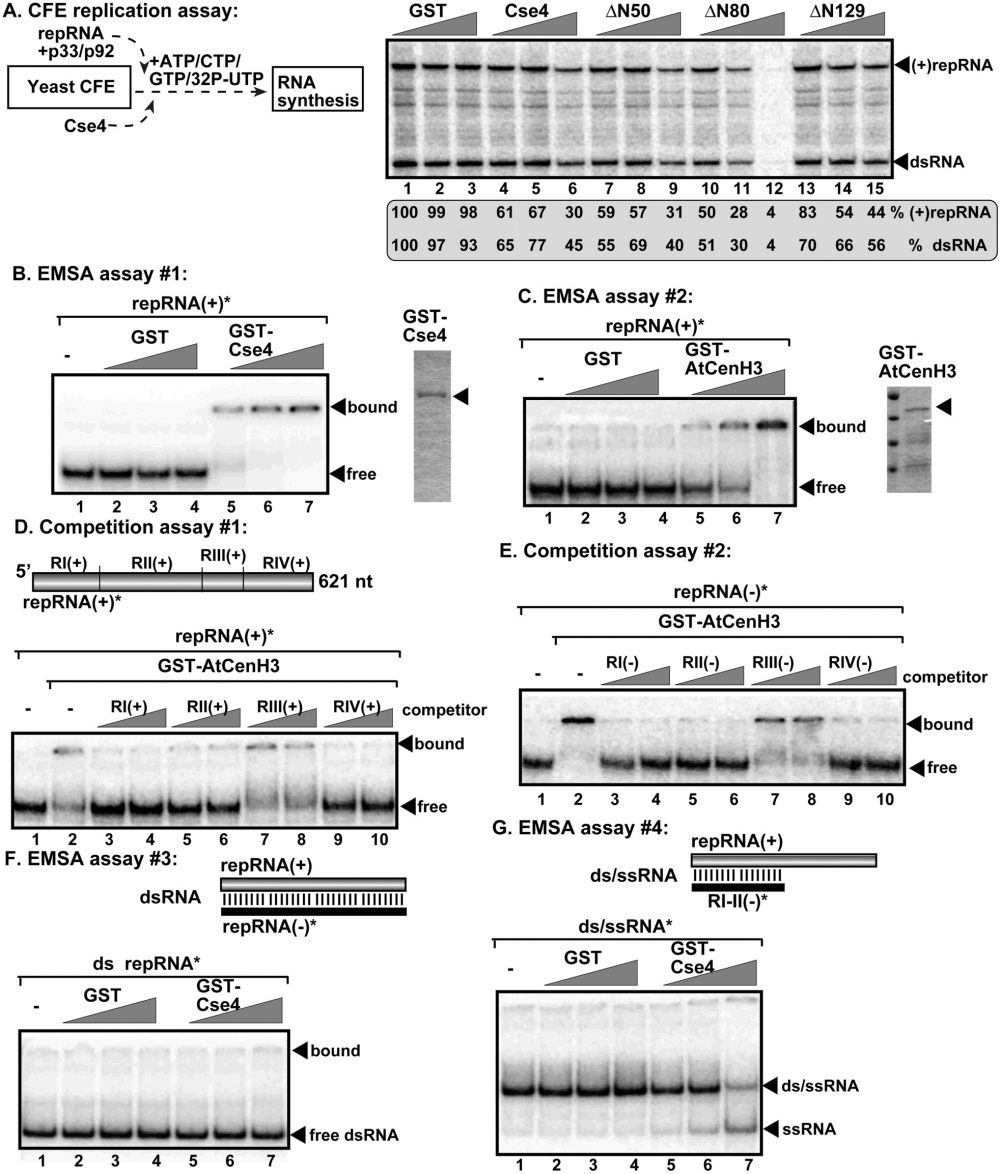
CenH3 has an RNA chaperone activity and inhibits tombusvirus replication *in vitro*. (A) Left panel: Scheme of the CFE replication assay. The CFE was prepared from WT yeast strain. Purified recombinant MBP-p33 and MBP-p92^pol^ TBSV replication proteins and *in vitro* transcribed TBSV DI-72 (+)repRNA were added to the CFE to reconstitute the active TBSV replicase. The affinity-purified recombinant WT GST-Cse4 (CenH3 homolog), Cse4 N-terminal deletion mutants and GST control were added to the assay as shown. Right panel: Denaturing PAGE analysis of the ^32^P-labeled TBSV repRNA products obtained in the CFE-based replication assay shows inhibition of TBSV replication by recombinant Cse4 or Cse4 mutants *in vitro*. (B, C) RNA gel mobility shift analysis shows that GST-Cse4 and GST-AtCenH3 bind to ^32^P-labeled (+)repRNA *in vitro*. Purified GST-Cse4, GST-AtCenH3 or GST were added in increasing concentrations (0.1, 0.2 or 0.4 μM) to the assays. The ^32^P-labeled ssRNA-protein complexes were visualized on nondenaturing 5% polyacrylamide gels. (D, E) RNA competition experiments. The assays contained 0.2 μM of purified GST or GST-AtCenH3 along with the ^32^P-labeled (+)repRNA or (-)repRNA templates (~0.1 pmol), and unlabeled competitor RNAs (2 and 4 pmol) representing one of the four regions of TBSV DI-72 RNA from both RNA strands (see panel D, top). The GST-AtCenH3—^32^P-labeled ssRNA complex was visualized on nondenaturing 5% acrylamide gels. (F, G) RNA-strand separation assays. Top: Schematic representation of the RNA/RNA duplexes used in the assays. The templates consist of DI-72 (+)repRNA and a ^32^P-labeled complementary (-)RNA creating a complete (F) or partial (G) RNA/RNA duplex. Bottom: Increasing amounts (0.1, 0.2 or 0.4 μM) of purified recombinant GST-Cse4 or GST (as a control), were added to the reactions. The ^32^P-labeled RNA products after the *in vitro* strand separation assay were analyzed on nondenaturing 5% polyacrylamide gels. Three or more independent repeats were performed for each experiment.

To test if the yeast Cse4 affects TBSV replication *in vitro*, we reconstituted the tombusvirus replicase by using (+)repRNA transcripts and purified recombinant TBSV p33 and p92^pol^ replication proteins in cell-free extracts (CFE) prepared from WT yeast (Fig 3A) [46, 52]. The affinity-purified recombinant yeast Cse4p was added in different amounts to the CFE-based replication assay at the beginning of the assay. At the end of the assay, we performed nondenaturing PAGE analysis of the *in vitro* replicase products. The replication assay revealed up to ~2-fold reduction in dsRNA replication intermediate and ~3-fold reduction in (+)ssRNA products in CFE with the highest amount of Cse4p in comparison with the RNA replication supported by WT CFE in the presence of GST control (Fig 3A, lanes 4–6 versus 1–3). The finding that both the new (-)RNA (present in dsRNA replication intermediate) [53] and the new (+)RNA products were decreased when CFE contained the highest Cse4p level suggests that Cse4p likely inhibits the TBSV replicase assembly steps, which occurs prior to (-)RNA and (+)RNA synthesis *in vitro*.

To identify the activity of CenH3 important for its viral restriction function, we tested if purified recombinant Cse4p or AtCenH3 could bind to the viral repRNA. Gel mobility shift assays with radiolabeled RNA probes showed that both Cse4p and AtCenH3 bound efficiently to the TBSV (+)RNA template *in vitro* (Fig 3B and 3C). Template competition assays revealed that three of the four regions of the repRNA with known *cis*-acting functions during TBSV replication [54–56] competed efficiently with (+)repRNA or (-)repRNA templates in vitro (Fig 3D and 3E). Because the secondary structures of these various regions are absolutely critical to support various steps in TBSV replication [57], we tested if CenH3 can modify double-stranded viral RNA structures. We found that Cse4p unwound partial dsRNA regions in the viral repRNA template in the absence of ATP (Fig 3G), whereas Cse4p was not efficient in separating complete dsRNA structure *in vitro* (Fig 3F). The ability of Cse4p to bind to the viral RNA and unwind a partial dsRNA template suggests that Cse4p functions as an RNA chaperone in TBSV replication *in vitro*.

To determine which domain of CenH3 is important to bind to the viral RNA and its chaperone function, we generated a series of truncation mutants of Cse4p, including the N-terminal domain involved in protein interactions and post-translation modifications (protein stability) and the C-terminal Histone-fold domain (HFD) containing the centromere targeting domain (CATD) [58]. Expression of two N-terminal deletion mutants (i. e., ΔN50 and ΔN80) in yeast inhibited TBSV repRNA accumulation to a similar extent as the full-length Cse4p (S2A Fig). Expression of ΔN129 led to low protein accumulation, suggesting the N-terminal region of Cse4p is needed for protein stability (S2A Fig). In contrast, expression of the Cse4p mutants lacking the highly conserved HFD domain (i. e., ΔC60 and ΔC100) in yeast did not inhibit TBSV repRNA accumulation (S2A Fig). The *in vitro* RNA binding experiments suggested that the N-terminal region in Cse4p is not required, whereas the C-terminal HFD domain is critical for Cse4p to bind to the TBSV repRNA (S2C and S2D Fig). RNA strand-separation experiments revealed that the mutants, similar to the full-length Cse4p, did not unwind a fully dsRNA structure (S2E Fig), whereas mutant ΔC100 was defective in separation of the partial ds/ssRNA structure, unlike the full-length and the N-terminal mutants (S2F Fig). Based on these experiments, we propose that the highly conserved HFD domain of Cse4p is involved in viral RNA binding and this domain also acts as an RNA chaperone on viral RNA templates.

To test if CenH3 can also interact with other viral components, we performed co-purification experiments from yeast co-expressing Flag-tagged AtCenH3 and His_6_-tagged p33 replication protein. After detergent-solubilization of the membrane-fraction of yeast, the Flag-CenH3 was immobilized to the Flag-column. Western blot analysis of the eluted proteins from the column revealed the co-purified His_6_-p33 (S3A Fig, lane 1). In a reverse co-purification experiment, we purified Flag-p33 from the detergent-solubilized membrane-fraction of yeast. The purified preparation also contained the Myc-tagged full-length Cse4p (S3B Fig, lane 2). Additional co-purification experiments revealed that the N-terminal fragment of Cse4p was not co-purified, whereas the C-terminal HFD domain of Cse4p was present in similarly purified Flag-p33 preparations (S3B Fig, lanes 3 and 4). These co-purification experiments demonstrated the interaction involving p33 replication protein and CenH3/Cse4p in the yeast membrane fraction.

To confirm direct interactions between TBSV p33 and Cse4p proteins in the absence of the viral RNA, we used a pull-down assay with the TBSV GST-tagged p33 and MBP-tagged Cse4p proteins from *E*. *coli* (S3C Fig). For the pull-down assay, we used truncated TBSV p33 protein missing its membrane-binding region to aid its solubility in *E*. *coli* (termed p33C, S3C Fig). The GST-based pull-down experiments suggested that the interaction between the p33 replication protein and Cse4p host protein occurs within the highly conserved HFD domain of Cse4p.

### Altering host gene expression by TBSV depends on CenH3 in yeast and plants

**Fig 4:**
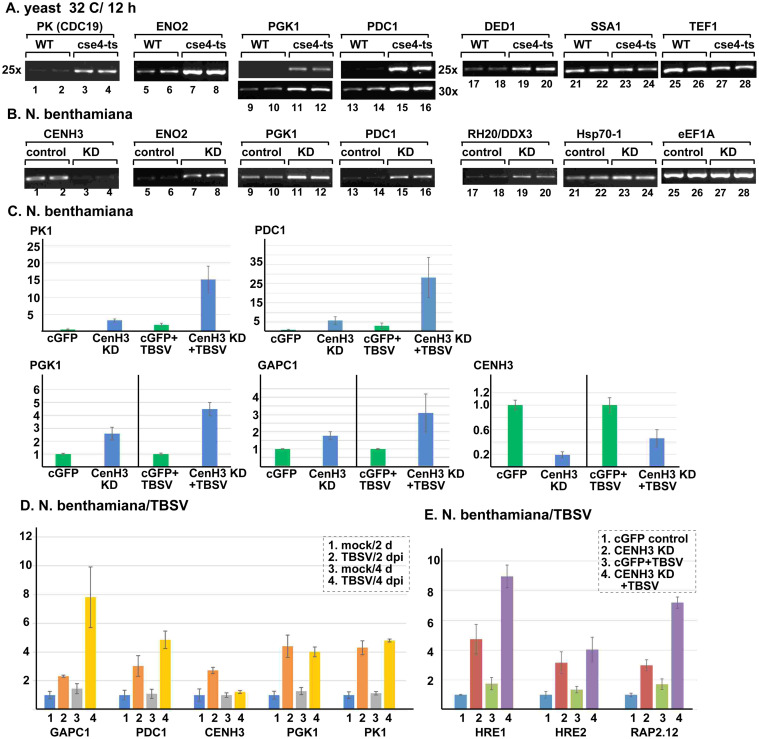
TBSV reprograms host gene expression via co-opting CenH3 in yeasts and plants. (A) Upregulation of mRNA expression of pro-viral host factors in cse4-1 mutant yeast. The mRNAs levels for glycolytic enzymes and other host proteins shown were estimated by semi-quantitative RT-PCR in total RNA samples obtained from WT or cse4-1 yeast cells grown at 32°C for 12 h. (B) Upregulation of mRNA expression of pro-viral host factors in CenH3-silenced *N*. *benthamiana* plants. The mRNA levels were estimated by semi-quantitative RT-PCR in total RNA samples obtained from either CenH3 knockdown or control plants (TRV-cGFP), 12 d after VIGS treatment. (C) Expression levels of mRNAs of *N*. *benthamiana* glycolytic and fermentation enzymes were estimated by real-time qRT-PCR in total RNA samples obtained from CenH3 knockdown (KD) or control plants (TRV-cGFP) in the absence or presence of TBSV replication. The mRNA level in TRV-cGFP control plants is chosen as 1 unit for each gene tested. (D) Expression levels of mRNAs of *N*. *benthamiana* glycolytic and fermentation enzymes were estimated by real-time qRT-PCR in total RNA samples obtained from either mock or TBSV inoculated plants, 2 (for inoculated leaves) or 4 (for systemic leaves) days post inoculation. (E) mRNAs expression levels of *N*. *benthamiana* hypoxia-related transcription factors were estimated by real-time qRT-PCR in the same total RNA samples as in panel C. Each experiment was repeated three times or more.

Previous work with cse4-1 yeast suggested that Cse4p has noncanonical functions in yeast outside of the centromeric area of the chromosome [33,34]. Cse4p acts as a noncanonical regulator of selected number of host genes via replacing histone molecules on the intergenic and promoter regions in chromosomal DNA [33,34]. Interestingly, genes whose expression is affected by Cse4p include several pro-viral host factors needed for robust TBSV replication. These host genes include several glycolytic and ethanol-producing enzymes [33,34], which are also selectively hijacked by TBSV into VROs to provide plentiful ATP locally to promote efficient TBSV replication [59–61]. Indeed, we have confirmed that mRNA expression for pyruvate kinase (PK, termed Cdc19 in yeast), Eno2 (Enolase 2), Pgk1 (phosphoglycerate kinase) and Pdc1 (pyruvate decarboxylase) glycolytic and fermentation enzymes and the pro-viral Ded1 DEAD-box helicase was increased in cse4-1 yeast at the semi-permissive temperature (Fig 4A). On the contrary, the expression of *SSA1* (Hsp70) and *TEF1* (eEF1A) genes has not changed in cse4-1 yeast at the semi-permissive temperature (Fig 4A). Ssa1 and Tef1 are key co-opted host proteins during TBSV replication in yeast [22,45,62,63]. The latter findings suggest that expression of a group of, but not all the pro-viral genes is affected by Cse4p in yeast.

VIGS-based knockdown of CenH3 level in *N*. *benthamiana* also led to enhanced expression of Eno2, Pgk1 and Pdc1 glycolytic/fermentation enzymes and the pro-viral RH20 (ortholog of the yeast Ded1) DEAD-box helicase (Fig 4B). Moreover, we have found that CenH3 knockdown in combination with TBSV infection of *N*. *benthamiana* led to the highest expression levels of PK1, Pgk1, GAPC1 (glyceraldehyde-3-phosphate dehydrogenase) and Pdc1 (Fig 4C). TBSV infection did also enhance the expression level of PK1, Pgk1, GAPC1 and Pdc1 by ~4-to-8-fold (Fig 4D). CenH3 expression was increased by ~3-fold at 2 dpi, followed by close to normal level of CenH3 expression at 4 dpi (Fig 4D). On the contrary, the expression of pro-viral Hsp70-1 and eEF1A plant genes [22,45,62,63] did not change in CenH3 knockdown plants (Fig 4B). Thus similar to the observations in yeast, the latter findings suggest that expression of a selective group of, but not all the pro-viral genes is affected by CenH3 in plants.

Because the fermentation enzymes (Adh1 and Pdc1) are induced during hypoxia (low O_2_ level due to plant submersion in water) by ERF-VII transcription factors in *Arabidopsis* plants, we measured the expression levels of RAP2.12, HRE1 and HRE2, which are known hypoxia transcription factors [64–66]. We have found that CenH3 knockdown in combination with TBSV infection of *N*. *benthamiana* led to the highest expression levels of RAP2.12, HRE1 and HRE2, which showed ~4-to-9-fold increase (Fig 4E). Interestingly, CenH3 knockdown or, separately, TBSV infection also enhanced the expression level of RAP2.12, HRE1 and HRE2 (Fig 4E).

These surprising findings on the shared function of TBSV infection and CenH3-based regulation of expression of glycolytic/fermentation enzymes and hypoxia-related transcription factors led to a new working model. The emerging idea is that TBSV hijacks CenH3/Cse4 from the nucleus into the VROs to affect the normal cellular gene-regulatory function of this conserved histone variant. This leads to alteration of gene expression of selected group of host genes whose expression is affected (directly or indirectly) by the noncanonical function of CenH3/Cse4.

### Regulation of expression of selected glycolytic and fermentation enzymes via CenH3 affects local ATP generation within TBSV VROs in yeast and plants

**Fig 5:**
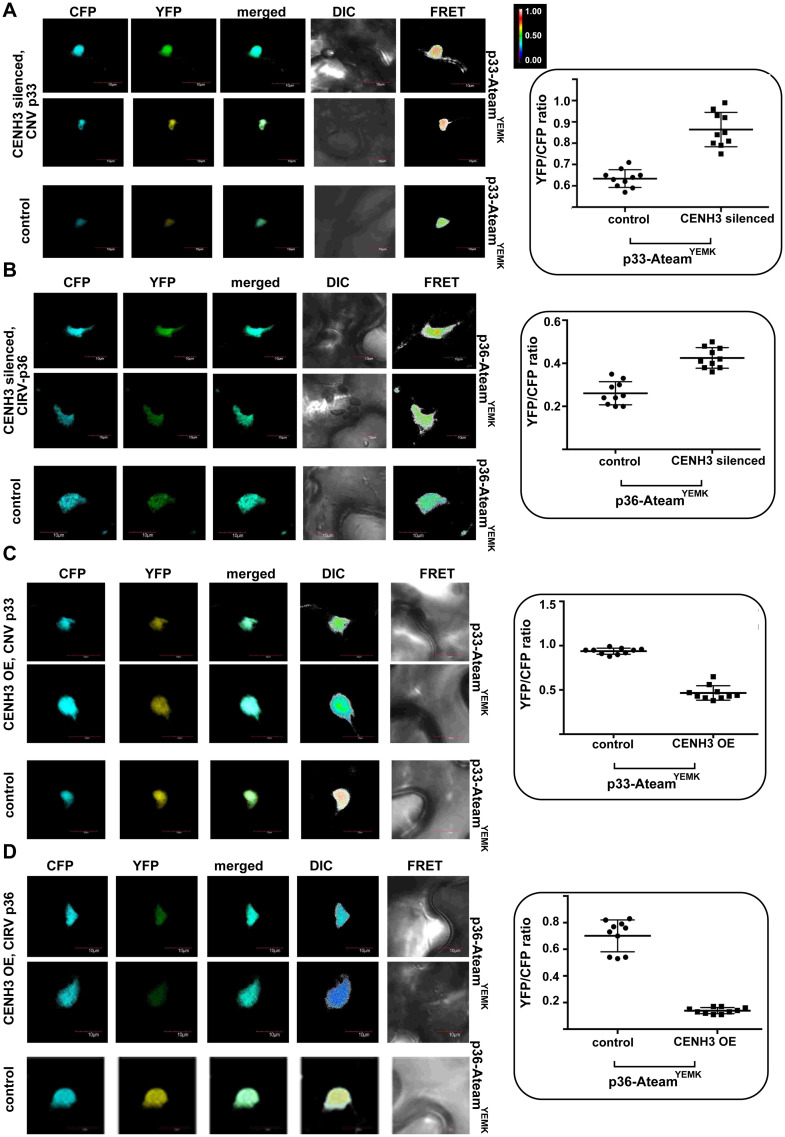
CenH3 expression level affects ATP production within tombusvirus VROs in plants. The ATP level within the VROs was measured using a FRET-based biosensor fused to the replication proteins. In this system ATP concentration is linearly correlated with the YFP:CFP ratio. Intense FRET signals (with high ratios between 0.5 to 1.0) are white and red, whereas weak FRET signals (ratios 0.1 and below) are dark and light blue. (A) VIGS-based knockdown of NbCenH3 was done as in Fig 1D. Eleven days later, co-expression of p33-ATeam^YEMK^, p92^pol^, repRNA and p19 suppressor of gene-silencing was done in upper leaves of *N*. *benthamiana* by agroinfiltration. Quantitative FRET values (obtained with ImageJ) for a number of samples are shown on the graph to the right. Top panels show CenH3-silenced plants, whereas the lower panel shows representative images obtained from non-silenced control plants. (B) Comparable experiments with NbCenH3 knockdown *N*. *benthamiana* plants co-expressing the mitochondria-associated CIRV p36-ATeam^YEMK^ and p95^pol^, repRNA and p19. See further details in (A). (C, D) ATP generation was measured in *N*. *benthamiana* plants where CenH3 was transiently over-expressed. Top panels: Confocal images are from CenH3 over-expression plants agroinfiltrated to co-express p33-ATeam^YEMK^, p92^pol^, repRNA and p19 (C) or to co-express p36-ATeam^YEMK^, p95^pol^, repRNA and p19 (D). The FRET signal measurements are shown on the right graph. Bottom panels: representative images obtained from control plants. Images and graphs are representative of two independent experiments.

To test the above model, we decided to measure ATP generation *in situ* within VROs, which depends on the availability of co-opted glycolytic and fermentation enzymes [67]. The ATP level within VROs was measured using a FRET-based biosensor [68], which we have used previously [60,61]. Briefly, we expressed p33-ATeam fusion protein in *N*. *benthamiana* leaves. The ATeam domain of the fusion protein can measure ATP level due to a conformational change in the enhanced ε subunit of the bacterial F_0_F_1_-ATP synthase upon ATP binding [60,61]. This is based on increased FRET signal in confocal laser microscopy when the ε subunit binds to ATP, resulting in a conformational change, which results in drawing the CFP and YFP fluorescent tags to close vicinity. On the other hand, the ATP-free form of the ε subunit is present in an extended conformation, which places CFP and YFP fluorescent tags in a distal position. This leads to low FRET signal [68]. We documented previously [60,61] that the p33-ATeam localizes to VROs representing aggregated peroxisomes. We found that the ATP level within VROs was ~40% higher in CenH3-silenced plants than in the control nonsilenced *N*. *benthamiana* plants (Fig 5A). Similar increased level of ATP production was detected within the CIRV-induced VROs using p36-ATeam fusion protein in CenH3-silenced *N*. *benthamiana* plants (Fig 5B). On the contrary, transient over-expression of CenH3 in *N*. *benthamiana* leaves reduced the ATP levels within CNV or CIRV-induced VROs by ~2- and ~4-fold, respectively (Fig 5C and 5D). Overall, the obtained data support the model that CenH3 is a critical host factor affecting the local ATP generation within the tombusvirus VROs in plant cells.

The effect of the orthologous Cse4p was also confirmed in yeast cells by using ATeam-p92^pol^ biosensor. We found previously [60,61] that the ATeam-tagged p92^pol^ is a fully functional RdRp, which localizes to VROs representing aggregated peroxisomes in yeast cells. Since these experiments are best performed in the presence of glucose in yeast medium [61], we used cse4-1 temperature sensitive mutant at semi-permissive 32°C in comparison with WT BY4741 strain. We detected increased production of ATP within VROs in cse4-1 strain in comparison with the WT yeast strain under the same growth conditions (S4 Fig). The emerging picture from the above experiments is that subversion of CenH3/Cse4p into VROs indirectly facilitates the more efficient ATP generation locally to support the energy requirement of virus replication.

## Discussion

Tombusviruses, similar to other (+)RNA viruses, exploit the host cells by co-opting the cellular translation machinery, subverting host proteins, intracellular membranes, metabolites and energy to build virus-induced extensive VROs in infected cells. It seems, however, that the molecular resources available in susceptible cells to support robust TBSV replication are suboptimal at the start of viral replication. Therefore, TBSV drives intensive remodeling and subversion of many cellular processes [42,69,70]. The virus-induced changes also include the dramatic alteration of gene expression in the nucleus. How the cytosolic TBSV accomplishes this feat is incompletely understood. The identification of the key role of the centromeric H3 variant in regulation of TBSV replication opens up a new page in TBSV-host interactions as discussed below.

### Is the nuclear CenH3/Cse4 histone variant a conventional viral restriction factor for the cytosolic tombusviruses?

Our gene and protein interaction network studies based on a dozen genome- and proteome-wide screens, which previously identified host components affecting TBSV replication and recombination or interactions with host components, revealed that Cse4 H3 histone variant is one of the highest connected nodes in the network [18]. Because Cse4/CenH3 (and the orthologous human CENP-A) is a nuclear protein with function within the centromere, it’s a puzzle how CenH3/Cse4 could be an important host factor for the cytosolic TBSV. However, yeast studies with a temperature-sensitive mutant of Cse4, over-expression or knockdown of the orthologous CenH3 in plants, all confirmed a strong restriction factor role for CenH3/Cse4 in tombusvirus replication. This conclusion with the peroxisome-associated TBSV is further supported by results obtained with the mitochondria-associated CIRV in yeast and plants.

How can a nuclear, DNA-binding histone variant be a restriction factor for a cytosolic RNA virus? Subcellular localization studies confirmed that CenH3/Cse4 is partially relocalized to the cytosol, namely into the large VROs, during TBSV or CIRV replication. Moreover, CenH3/Cse4 binds to the viral RNA *in vitro* via its HFD domain. We also showed RNA chaperone activity for Cse4 *in vitro*, which activity might contribute to the inhibitory function of Cse4 via unwinding critical *cis*-acting elements in the viral (+)RNA. We also documented interaction of CenH3/Cse4 with the p33 replication protein, which completely overlaps with the N-terminal region of the p92 RdRp. All these results agree with a proposed antiviral activity of the CenH3/Cse4. Accordingly, we have shown in this paper that the purified recombinant Cse4p is indeed inhibitory to TBSV replication in an *in vitro* replicase reconstitution assay.

### Recruitment of CenH3/Cse4 into VROs leads to selective reprogramming of host gene transcription during tombusvirus replication

Our initial results in yeast, plants and *in vitro* were not consistent with the idea that the canonical function of CenH3/Cse4 is exploited by the host to fight off tombusvirus infection. Moreover, tombusviruses replicate in mature plant leaf and root cells, which are not going through cell division and chromosomal segregation. An interesting noncanonical function of Cse4 is to replace histones bound to the chromosome in many places, including transcription factor hotspots [33–36]. In this role, Cse4 acts as a negative regulator of gene expression of over a hundred yeast genes [33–35]. Because these genes also include critical host factors, such as glycolytic enzymes, with pro-tombusvirus functions, we propose the idea that tombusviruses subvert CenH3/Cse4 into VROs to alter the gene expression in the nucleus. We propose that the TBSV-driven subversion of CenH3/Cse4 and partial sequestration into VROs have two major consequences for tombusvirus replication: (i) Indirectly altering host gene transcription in the nucleus. Accordingly, we show that similar to knocking down/inhibiting CenH3/Cse4 activities, TBSV infection also increased the expression of selected host genes, namely glycolytic and fermentation enzymes and others. This in turn helps TBSV to recruit pro-viral host factors from the more abundant protein pool, which is the consequence of sequestered CenH3/Cse4 on select gene expression. Altogether, these activities led to increased generation of ATP locally within VROs (Fig 5) likely due to the efficient recruitment of glycolytic and fermentation enzymes. *In situ* production of ATP within VROs is essential for robust TBSV replication as shown previously [61,67]. On the other hand, over-expression of CenH3/Cse4 might interfere with the TBSV-driven efficient sequestration of this host factor from the nucleus. Indeed, over-expression of CenH3/Cse4 inhibited local ATP generation within VROs and strongly inhibited TBSV replication. (ii) The second direct consequence of subverting CenH3/Cse4 to the VROs from the nucleus is that this process is “costly” to TBSV. This is because the virus must dedicate viral components, namely portion of the viral (+)RNA population and p33 molecules to subvert CenH3/Cse4 from the nucleus. A lower level of CenH3/Cse4 in the cell via mutation or depletion might help TBSV commit less of its components to sequester CenH3/Cse4 away from the nucleus. Accordingly, depletion or mutation in CenH3/Cse4 led to highly enhanced TBSV and CIRV replication in yeast and plant cells. On the contrary, over-expression of CenH3/Cse4 would force TBSV to commit even more viral components for sequestration (i.e., taking the viral (+)RNA and p33 away from replication functions), thus leading to reduced viral replication. We propose that this sequestration process of CenH3/Cse4 via TBSV components renders CenH3/Cse4 functioning as a restriction factor under given circumstances in *N*. *benthamiana* and yeast. Albeit CenH3 acts as a restriction factor of TBSV replication in *N*. *benthamiana* and yeast, one can imagine that CenH3 might act as pro-viral in other hosts. However, demonstration of this scenario requires future studies.

Hijacking and regulating CenH3 function might be conducted by other viruses as well. For example, Hepatitis B virus x protein (HBx) induces hepatocellular carcinoma (HCC) by inducing the over-expression of CENP-A (CenH3) protein [71]. The hepatitis C virus (HCV)-related chronic liver disease also correlates with increased level of CENP-A expression [72]. Interestingly, the NS1 protein of the influenza A H3N2 subtype contains a histone H3-like sequence (mimicking H3 structure), which is used to hijack host proteins [73].

### Summary

CenH3/Cse4 histone variant plays a major role in tombusvirus replication in plants and in yeast model host. The emerging theme from our current studies is that sequestration of CenH3/Cse4 from the nucleus into the cytosolic VROs by tombusviruses requires a balancing act between restriction and pro-viral activities. Robust TBSV replication depends on the virus’ ability to reprogram host gene transcription, in which CenH3/Cse4 plays a mostly unexplored role. Subversion of CenH3/Cse4 is a “double-edge sword”: possibly advantageous for TBSV under given conditions, but disadvantageous under over-expression conditions, when CenH3/Cse4 acts as a strong restriction factor. Our data indicate that the nuclear CenH3 histone variant plays surprising novel pro-viral and restriction functions during tombusvirus replication. Nevertheless, the results point at a new frontier in cytosolic tombusvirus—host interactions.

## Materials and methods

### Yeast strains and expression plasmids

*Saccharomyces cerevisiae* strains BY4741 and R1158 (wt) [74] were obtained from Open Biosystems. See further details in the supplementary materials (S1 Text).

### Analysis of tombusvirus replication in yeast

For measuring TBSV repRNA accumulation, yeast strains BY4741 and cse4-1 were transformed with HpGBK-CUP1-Hisp-33/ADH1-DI-72 and LpGAD-CUP1-His-p92. Two sets of cultures per strain were grown at 23°C 12 h overnight in SC-LH^-^ (synthetic complete medium without leucine and histidine) medium containing 2% glucose and 100 μM BCS (bathocuproinedisulfonic acid). Then, cells were centrifuged and washed thoroughly with clean SC-LH^-^ supplemented with 2% glucose medium, to remove BCS, and pellets were re-suspended in the same medium containing 50 μM CuSO_4_ to induce viral protein expression and repRNA replication. Then, a set of cultures was placed at 32°C and the other set remained at 23°C and grown for additional 24 h, time after which, total RNA and protein was extracted. For CIRV repRNA accumulation cells were co-transformed with plasmids HpESC-GAL1-Hisp36/GAL10-DI-72 and LpESC-CUP1-Flag-CIRVp95 and grown as above with the exception that pellets were thoroughly washed with SC-LH^-^ medium (plus 2% galactose) and re-suspended in SC-LH^-^ medium containing 2% galactose and 50 μM CuSO_4_. Total RNA and protein was extracted after 30 h of viral induction.

For over-expression analysis, BY4741 cells were transformed with plasmids HpGBK-CUP1-Hisp-33/ADH1-DI-72, LpGAD-CUP1-His-p92 and either UpESC empty vector, UpESC-HisAtCENH3, or pGAL-myc-CSE4. Transformed cells were grown for 24 h in SC-ULH^-^ medium supplemented with 2% galactose and 100 μM BCS. Cells then were centrifuged and washed thoroughly with clean SC-ULH^-^ medium supplemented with 2% galactose and resuspended in the same medium containing 50 μM CuSO_4_ to induce viral repRNA replication. Total RNA and protein were isolated after 24 h. The same method was used when expressing the Cse4 truncation mutants, but BY471 yeast cells were transformed with HpGBK-CUP1-Hisp-33/ADH1-DI-72, LpGAD-CUP1-His-p92 and either UpYES empty vector, UpYES-His-CSE4, UpYES-His-cse4ΔN50, UpYES-His-cse4ΔN80, UpYES-His-cse4ΔN129, UpYES-His-cse4ΔC60, UpYES-His-cse4ΔC100 or UpYES-His-Histone H3.

### Tombusvirus replication assay in *N*. *benthamiana* plants

The virus-induced gene silencing (VIGS) in *N*. *benthamiana* was done as described previously [50,75]. After 11 d of VIGS treatment (pTRV1 together with pTRV2-5’CENH3, pTRV2-3’CENH3-3’ or pTRV2-cGFP control) two distal leaves were sap inoculated with TBSV or CIRV virions. Samples were collected 2 d post-infection (dpi) for TBSV-infected leaves or 3 dpi from CIRV-infected leaves. Viral RNA accumulation was analyzed by northern blot after total RNA extraction. Silencing was confirmed by RT-PCR with primers oligo-d(T) (for RT) and #6380/6381 or #6382/6383, (for PCR) to detect CENH3 mRNA or primers #2859/#2860 to detect tubulin mRNA as RT-PCR amplification control.

To over-express CenH3, *N*. *benthamiana* leaves were co-infiltrated with *Agrobacterium* cultures containing pGD-p19 and either pGD empty vector, pGD-NbCENH3 or pGD-AtCENH3. In the experiment with CNV, plants were also infiltrated with *Agrobacterium* carrying pGD-CNV^20KSTOP^ 24 h after the first agroinfiltration. In the experiment with TBSV and CIRV, plants were inoculated with crude sap inoculum 48 h after agroinfiltration. Samples were collected from CNV infiltrated leaves about 84 h after the second agroinfiltration. For TBSV and CIRV infection, samples were taken from inoculated leaves 48 h and 72 h post-virus inoculation, respectively. All samples were used for total RNA extraction and northern blot as described, to analyze the accumulation levels of these viruses [76].

### Confocal laser scanning microscopy

To observe the subcellular localization of CenH3 in *N*. *benthamiana* epidermal cells, transgenic *N*. *benthamiana* (constitutively expressing H2B fused to RFP) leaves were agroinfiltrated with plasmids pGD-T33-BFP or pGD-C36-BFP (OD_600_ 0.3), pGD-p19 (OD_600_ 0.3) and pGD-GFP-NbCENH3 (OD_600_ 0.3). Likewise, wild-type *N*. *benthamiana* leaves were agroinfiltrated with plasmids pGD-T33-BFP or pGD-C36-BFP (OD_600_ 0.25), pGD-p19 (OD_600_ 0.25), pGD-GFP-NbCENH3 (OD_600_ 0.25) and either pGD-RFP-SKL (OD_600_ 0.25) or pGD-Cox4-RFP (OD_600_ 0.25). In the experiment with CNV, plants were also infiltrated with *Agrobacterium* carrying pGD-CNV^20KSTOP^ (OD_600_ 0.2). Plants were additionally inoculated with TBSV or CIRV virions. Live confocal images were obtained with an Olympus FV1000 microscope (Olympus America) 48 h for CNV and TBSV-infection and 72 h for CIRV- infection. BFP/Alexa 405, GFP/Alexa 488, and RFP were excited using 405 nm, 488 nm, or 543 nm lasers, respectively. Images were obtained sequentially and merged using Olympus FLUOVIEW 1.5 software [77].

For confocal microscopy assays in yeast, BY4741 cells were co-transformed with plasmids UpYES-GAL-YFP-CSE4, LpGAD and HpESC empty vectors or UpYES-GAL-YFP-CSE4, LpGAD-CUP1-His-p92 and HpESC-GAL1-CFP-p33/GAL1-DI72. Transformed yeast cell cultures were grown 12 h overnight in the proper SC-ULH^-^ supplemented with 2% galactose and 100 μM BCS. Next morning, cells were centrifuged and washed (with sterile water) and pellets were re-suspended in the same medium supplemented with 2% galactose and 50 μM CuSO_4_ to induce the expression of the fluorescently-tagged proteins. Samples were collected at the time points given in the main figure and analyzed by confocal microscopy as previously described [78].

### Estimation of mRNA expression levels for selected host factors

For the mRNA detection in yeast, BY4741 and cse4-1 cells were grown for 12 h at 32°C. Total RNA was isolated and analyzed by gel electrophoresis in a 1.5% agarose gel to normalize total RNA levels in the samples. Then, the same amounts of total RNA were used for semi-quantitative RT-PCR reactions. cDNA was first obtained using MMLV reverse transcriptase (Lucigen) and oligo dT. The cDNA was then used to perform PCR to detect the expression levels of the following host factors with the following primer sets: #5992/#7136 for Cdc19; #7123/#7137 for Eno2; #6275/#6367 for Pgk1; #5621/#5604 for Pdc1; #4308/#7140 for Ded1; #2030/#7138 for Ssa1 and #7141/#7142 for Tef1.

For mRNA detection in plant, a similar approach was performed by using wild-type and CenH3 knockdown *N*. *benthamiana* leaf samples. VIGS was performed as described using the pTRV2-3’CENH3-3’ construct only. After 12 d of VIGS treatment, total RNA was isolated and used for semi-quantitative RT-PCR using the following primers: #6380/#6381 for CenH3; #7293/#7294 for Eno2; #7291/#7292 for Pgk1; #7289/#7290 for Pdc1; #7295/#7296 for RH20 (DDX3-like); #2534/#2535 for Hsp70-1 and #7297/#7298 for eEF1A.

Real-Time quantitative qRT-PCR was also used for the detection of *N*. *benthamiana* gene expression as follows: VIGS was performed as described using the pTRV2-3’CENH3-3’ construct only. After 11 d of VIGS treatment, plants were inoculated with TBSV virions or mock inoculated. Samples were collected 2 d post-inoculation from infected leaves and 4 d post-inoculation from systemically-infected leaves. Total RNA was isolated and used for qRT-PCR. First, primers were designed using Real Time qPCR Tool from Integrated DNA Technologies website (https://www.idtdna.com/scitools/Applications/RealTimePCR/). Second, MMLV reverse transcriptase (Lucigen) and Oligo-dT were used to obtain cDNA. Finally, the qPCR reactions were prepared using Applied Biosystem Power UP SYBR green master mix (Thermo Fisher Scientific) in a 96 well plate and the Eppendorf’s Mastercycler ep realplex instrument and primers #8217/#8218 for GapC1; #8219/#8220 for Pgk1; #8221/#8222 for CenH3; #8174/#8175 for Pdc1; #8176/#8177 for PK1 and #8178/#8179 for Tubulin β2 as the housekeeping gene control. qPCR conditions were selected following the Power Up SYBR green master mix user manual recommendations.

### Visualization and measurement of ATP levels within VROs in plants

Intracellular ATP levels within VROs were visualized using the ATeam-based biosensor [68] by using a confocal microscope and measured by FRET analysis. To detect the ATP levels within the tombusvirus VROs in CenH3-silenced or control *N*. *benthamiana* plants, leaves were co-agroinfiltrated with plasmids pGD-p33-ATeam^YEMK^, pGD-p92, pGD-DI-72 and pGD-p19 for CNV infection or plasmids pGD-p36-ATeam^YEMK^, pGD-p95, pGD-DI-72 and pGD-p19 for CIRV infection. Samples were analyzed in a confocal microscope 2 d post-agroinfiltration. FRET images were obtained by exciting cells with a 405-nm laser diode, and CFP and YFP (Venus) signals were detected at 480–500 nm and 515–615 nm wavelength ranges, respectively [61]. Each YFP/CFP ratio was calculated by dividing pixel-by-pixel a Venus image with a CFP image using Olympus FLUOVIEW software and ImageJ software. In the case of CenH3 over-expression experiments, *N*. *benthamiana* leaves were co-infiltrated with the above plasmid combinations for CNV and CIRV with the addition of pGD-NbCENH3 plasmid as well. Confocal FRET images were obtained as above, 2.5 d post-agroinfiltration.

## Supporting information

S1 Text Supplementary materials and methods. (DOCX)

S1 Fig Re-distribution of nuclear Cse4/CenH3 to the sites of viral replication in yeast. Confocal laser microscopy analyses in WT yeast cells co-expressing YFP-Cse4 together with CFP-p33, p92^pol^ replication proteins and the (+)repRNA show partial co-localization of YFP-Cse4 with CFP-p33 at 12 h, 16 h and 24 h after induction of protein expression. Images on the right show the nuclear distribution of YFP-Cse4 in the absence of viral components in WT yeast cells at the same time points.
(TIF)

S2 Fig The highly conserved HFD of Cse4p is involved in viral RNA binding and RNA chaperone activity. (A) Schematic diagram showing the endpoints of the Cse4 deletion mutants used in this study. The proteins were named by the number of the last amino acid deleted. (B) Top: northern blot analysis shows a reduction in the accumulation of repRNA in cells expressing full length Cse4 (lanes 3–4) and the N-terminal deletion mutants ΔN50 (lanes 5–6) and ΔN80 (lanes 7–8) compared to the control samples (lanes 1–2). Expression of ΔN129, ΔC60, ΔC100 and Histone H3 did not affect TBSV repRNA accumulation (lanes 9–16). Middle: Northern blot with 18S ribosomal RNA specific probe was used as a loading control. Bottom: Western blot analyses of the level of His_6_-p33 replication protein and His_6_-Cse4 mutants with anti-His antibody. Note that levels of ΔN129 are very low, suggesting that the N-terminal region of Cse4 has a role in protein stability. (C, D) RNA gel mobility shift analysis shows that GST-Cse4, GST-ΔN50 and GST-ΔN80 efficiently bind to ^32^P-labeled (+)repRNA (C) or (-)repRNA (D) *in vitro*, whereas GST-ΔC100 show defective binding capability to both repRNAs. Purified GST-Cse4, GST-ΔN50, GST-ΔN80, GST-ΔC100 and GST were added in increasing concentrations (0.1, 0.2 or 0.4 μM) to the assays. The ^32^P-labeled ssRNA—protein complexes were visualized on nondenaturing 5% polyacrylamide gels. (E, F) RNA-strand separation assays. Left: Schematic representation of the RNA/RNA duplexes used in the assays. See details in Fig 3F and 3G. Right: Increasing amounts (0.1, 0.2 or 0.4 μM) of purified recombinant GST-Cse4, GST-ΔN50, GST-ΔN80, GST-ΔC100 and GST (as a control), were added to the assays. The ^32^P-labeled RNA products after the *in vitro* RNA-strand separation assay were analyzed on nondenaturing 5% polyacrylamide gels. Full length Cse4 and all mutants were unable to unwind a fully duplexed dsRNA (E), whereas WT Cse4 and the N-terminal deletion mutants unwound the partial dsRNA (F). Three independent repeats were performed for each experiment.
(TIF)

S3 Fig CenH3 interacts with TBSV p33 replication protein. (A) Co-purification of viral p33 replication protein with plant CenH3. First panel: western blot analysis of co-purified His_6_-p33 with Flag-affinity purified *A*. *thaliana* Flag-CenH3 from membrane fraction of WT yeast. His_6_-p33 was detected with anti-His antibody. The negative control was His_6_-tagged AtCenH3 which was not co-purified from yeast extracts when using a Flag-affinity column (lane 2). Second panel: Western blot of purified Flag-AtCenH3 and Flag-p33 detected with anti-Flag antibody. Bottom panels: Western blot of His_6_- or Flag-tagged proteins in total yeast extracts. (B) Co-purification of yeast Cse4p with the viral replicase complex. First panels: western blot analysis of co-purified Myc-tagged Cse4 (lane 2) and Cse4 C-terminal domain (Cse4-CTD, lane 4) with Flag-affinity purified Flag-p33 from WT yeast membrane fraction. Myc-Cse4 and Myc-Cse4-CTD were detected with anti-Myc antibody. The negative control was His_6_-tagged p33 (lane 1). Second panels: Western blots of purified Flag-p33 detected with anti-Flag antibody. Bottom panels: Western blot of Myc-Cse4, Myc-Cse4-CTD, Myc-Cse4 N terminal domain (Myc-Cse4-N) and Flag-tagged p33 in the total yeast extracts. Note that after affinity-purification, Myc-Cse4-N was not co-purified with p33 (lane 3). (C) Pull-down assay including TBSV GST-p33 replication protein and the MBP-tagged Cse4 or Cse4 deletion mutants. The C-terminal region of TBSV p33 replication protein was used instead of the full-length protein, which includes the non-soluble N-terminal region with the trans-membrane domains. Top: Western blot analysis of the captured GST-p33C with MBP purified WT GST-Cse4 or ΔN50, ΔN80, ΔN129 Cse4 deletion mutants. GST was used as a control. Note that similar to Myc-Cse4-N, the C-terminal deletion mutants ΔC60 and ΔC100 were not pulled-down with GST-p33C suggesting that the HFD of Cse4 is also important for the interaction between p33 replication protein and Cse4p. Bottom: Coomassie-blue stained SDS-PAGE of the purified recombinant proteins. All experiments were performed three times.
(TIF)

S4 Fig Reprogramming of glycolytic and fermentation enzymes expression by TBSV via CenH3 affects ATP accumulation within VROs in yeast. Comparison of the ATP level within the tombusvirus replication compartment in WT and cse4-1 yeasts grown at 23°C using ATeam^YEMK^–p92^pol^. See further details in Fig 5. Increased generation of ATP within VROs was observed in cse4-1 temperature sensitive strain compared to control WT yeast grown under the same conditions. White dashed lines separate two yeast cells. Images are representative of two independent experiments.
(TIF)

## References

[1] Nagy PD, Pogany J. The dependence of viral RNA replication on co-opted host factors. Nature Reviews Microbiology. 2012;10(2):137–49. ISI:000299115000013. doi: 10.1038/Nrmicro2692 22183253 PMC7097227

[2] Belov GA, van Kuppeveld FJ. (+)RNA viruses rewire cellular pathways to build replication organelles. Curr Opin Virol. 2012;2(6):740–7. Epub 2012/10/06. S1879-6257(12)00141-1 [pii] doi: 10.1016/j.coviro.2012.09.006 . 23036609 PMC7102821

[3] Shulla A, Randall G. Hepatitis C virus-host interactions, replication, and viral assembly. Curr Opin Virol. 2012;2(6):725–32. Epub 2012/10/23. S1879-6257(12)00159-9 [pii] doi: 10.1016/j.coviro.2012.09.013 . 23083892 PMC3508086

[4] Wang A. Dissecting the molecular network of virus-plant interactions: the complex roles of host factors. Annu Rev Phytopathol. 2015;53:45–66. Epub 2015/05/06. doi: 10.1146/annurev-phyto-080614-120001 . 25938276

[5] de Wilde AH, Snijder EJ, Kikkert M, van Hemert MJ. Host Factors in Coronavirus Replication. Curr Top Microbiol Immunol. 2018;419:1–42. Epub 2017/06/24. doi: 10.1007/82_2017_25 . 28643204 PMC7119980

[6] Sanfacon H. Plant Translation Factors and Virus Resistance. Viruses. 2015;7(7):3392–419. Epub 2015/06/27. v7072778 [pii] doi: 10.3390/v7072778 . 26114476 PMC4517107

[7] Hyodo K, Okuno T. Hijacking of host cellular components as proviral factors by plant-infecting viruses. Advances in virus research. 2020;107:37–86. doi: 10.1016/bs.aivir.2020.04.002 32711734

[8] Garcia-Ruiz H. Susceptibility genes to plant viruses. Viruses. 2018;10(9):484. doi: 10.3390/v10090484 30201857 PMC6164914

[9] den Boon JA, Ahlquist P. Organelle-like membrane compartmentalization of positive-strand RNA virus replication factories. Annu Rev Microbiol. 2010;64:241–56. Epub 2010/09/10. doi: 10.1146/annurev.micro.112408.134012 . 20825348

[10] Nagy PD. Host protein chaperones, RNA helicases and the ubiquitin network highlight the arms race for resources between tombusviruses and their hosts. Adv Virus Res. 2020;107:133–58. Epub 2020/07/28. S0065-3527(20)30024-5 [pii] doi: 10.1016/bs.aivir.2020.06.006 . 32711728 PMC7342006

[11] Nagy PD. Tombusvirus-Host Interactions: Co-Opted Evolutionarily Conserved Host Factors Take Center Court. Annu Rev Virol. 2016;3(1):491–515. Epub 2016/09/01. doi: 10.1146/annurev-virology-110615-042312 . 27578441

[12] Sasvari Z, Lin W, Inaba JI, Xu K, Kovalev N, Nagy PD. Co-opted Cellular Sac1 Lipid Phosphatase and PI(4)P Phosphoinositide Are Key Host Factors during the Biogenesis of the Tombusvirus Replication Compartment. J Virol. 2020;94(12). Epub 2020/04/10. JVI.01979-19 [pii] doi: 10.1128/JVI.01979-19 . 32269127 PMC7307105

[13] Nagy PD, Pogany J. Global genomics and proteomics approaches to identify host factors as targets to induce resistance against Tomato bushy stunt virus. Adv Virus Res. 2010;76:123–77. Epub 2010/10/23. S0065-3527(10)76004-8 [pii] doi: 10.1016/S0065-3527(10)76004-8 . 20965073 PMC7173251

[14] Nagy PD. Yeast as a model host to explore plant virus-host interactions. Annu Rev Phytopathol. 2008;46:217–42. Epub 2008/04/22. doi: 10.1146/annurev.phyto.121407.093958 . 18422427

[15] Nagy PD, Barajas D, Pogany J. Host factors with regulatory roles in tombusvirus replication. Curr Opin Virol. 2012;2(6):685–92. Epub 2012/11/06. S1879-6257(12)00165-4 [pii] doi: 10.1016/j.coviro.2012.10.004 . 23122856

[16] Li Z, Nagy PD. Diverse roles of host RNA binding proteins in RNA virus replication. RNA Biol. 2011;8(2):305–15. Epub 2011/04/21. 15391 [pii]. doi: 10.4161/rna.8.2.15391 . 21505273 PMC3230553

[17] Nawaz-ul-Rehman MS, Reddisiva Prasanth K, Baker J, Nagy PD. Yeast screens for host factors in positive-strand RNA virus replication based on a library of temperature-sensitive mutants. Methods. 2013;59(2):207–16. Epub 2012/11/14. S1046-2023(12)00279-4 [pii] doi: 10.1016/j.ymeth.2012.11.001 . 23147170

[18] Sasvari Z, Alatriste Gonzalez P, Nagy PD. Tombusvirus-yeast interactions identify conserved cell-intrinsic viral restriction factors. Front Plant Sci. 2014;5:383. Epub 2014/08/27. doi: 10.3389/fpls.2014.00383 . 25157258 PMC4127529

[19] Nagy PD. Exploitation of a surrogate host, Saccharomyces cerevisiae, to identify cellular targets and develop novel antiviral approaches. Curr Opin Virol. 2017;26:132–40. Epub 2017/08/27. S1879-6257(17)30102-5 [pii] doi: 10.1016/j.coviro.2017.07.031 . 28843111

[20] Shah Nawaz-ul-Rehman M, Martinez-Ochoa N, Pascal H, Sasvari Z, Herbst C, Xu K, et al. Proteome-wide overexpression of host proteins for identification of factors affecting tombusvirus RNA replication: an inhibitory role of protein kinase C. J Virol. 2012;86(17):9384–95. Epub 2012/06/22. JVI.00019-12 [pii] doi: 10.1128/JVI.00019-12 . 22718827 PMC3416130

[21] Mendu V, Chiu M, Barajas D, Li Z, Nagy PD. Cpr1 cyclophilin and Ess1 parvulin prolyl isomerases interact with the tombusvirus replication protein and inhibit viral replication in yeast model host. Virology. 2010;406(2):342–51. Epub 2010/08/17. S0042-6822(10)00475-7 [pii] doi: 10.1016/j.virol.2010.07.022 . 20709345

[22] Li Z, Pogany J, Panavas T, Xu K, Esposito AM, Kinzy TG, et al. Translation elongation factor 1A is a component of the tombusvirus replicase complex and affects the stability of the p33 replication co-factor. Virology. 2009;385(1):245–60. Epub 2009/01/10. S0042-6822(08)00791-5 [pii] doi: 10.1016/j.virol.2008.11.041 . 19131084 PMC2785496

[23] Li Z, Barajas D, Panavas T, Herbst DA, Nagy PD. Cdc34p ubiquitin-conjugating enzyme is a component of the tombusvirus replicase complex and ubiquitinates p33 replication protein. J Virol. 2008;82(14):6911–26. Epub 2008/05/09. JVI.00702-08 [pii] doi: 10.1128/JVI.00702-08 . 18463149 PMC2446948

[24] Panavas T, Serviene E, Brasher J, Nagy PD. Yeast genome-wide screen reveals dissimilar sets of host genes affecting replication of RNA viruses. Proc Natl Acad Sci U S A. 2005;102(20):7326–31. Epub 2005/05/11. 0502604102 [pii] doi: 10.1073/pnas.0502604102 . 15883361 PMC1129141

[25] Ding Y, Lozano-Durán R. The Cajal Body in Plant-Virus Interactions. Viruses. 2020;12(2). Epub 2020/02/28. doi: 10.3390/v12020250 . 32102236 PMC7077289

[26] Lopez-Denman AJ, Mackenzie JM. The IMPORTance of the Nucleus during Flavivirus Replication. Viruses. 2017;9(1). Epub 2017/01/21. doi: 10.3390/v9010014 . 28106839 PMC5294983

[27] Shaw J, Yu C, Makhotenko AV, Makarova SS, Love AJ, Kalinina NO, et al. Interaction of a plant virus protein with the signature Cajal body protein coilin facilitates salicylic acid-mediated plant defence responses. New Phytol. 2019;224(1):439–53. Epub 2019/06/20. doi: 10.1111/nph.15994 . 31215645

[28] Yarbrough ML, Mata MA, Sakthivel R, Fontoura BM. Viral subversion of nucleocytoplasmic trafficking. Traffic. 2014;15(2):127–40. Epub 2013/12/03. doi: 10.1111/tra.12137 . 24289861 PMC3910510

[29] Wieland G, Orthaus S, Ohndorf S, Diekmann S, Hemmerich P. Functional complementation of human centromere protein A (CENP-A) by Cse4p from Saccharomyces cerevisiae. Mol Cell Biol. 2004;24(15):6620–30. Epub 2004/07/16. [pii]. doi: 10.1128/MCB.24.15.6620-6630.2004 . 15254229 PMC444843

[30] Quenet D, Dalal Y. A long non-coding RNA is required for targeting centromeric protein A to the human centromere. Elife. 2014;3:e03254. Epub 2014/08/15. doi: 10.7554/eLife.03254 . 25117489 PMC4145801

[31] Corless S, Hocker S, Erhardt S. Centromeric RNA and Its Function at and Beyond Centromeric Chromatin. J Mol Biol. 2020;432(15):4257–69. Epub 20200402. doi: 10.1016/j.jmb.2020.03.027 . 32247764

[32] Nechemia-Arbely Y, Miga KH, Shoshani O, Aslanian A, McMahon MA, Lee AY, et al. DNA replication acts as an error correction mechanism to maintain centromere identity by restricting CENP-A to centromeres. Nat Cell Biol. 2019;21(6):743–54. Epub 20190603. doi: 10.1038/s41556-019-0331-4 . 31160708 PMC7015266

[33] Lefrancois P, Euskirchen GM, Auerbach RK, Rozowsky J, Gibson T, Yellman CM, et al. Efficient yeast ChIP-Seq using multiplex short-read DNA sequencing. BMC Genomics. 2009;10:37. Epub 2009/01/23. 1471-2164-10-37 [pii] doi: 10.1186/1471-2164-10-37 . 19159457 PMC2656530

[34] Lefrancois P, Auerbach RK, Yellman CM, Roeder GS, Snyder M. Centromere-like regions in the budding yeast genome. PLoS Genet. 2013;9(1):e1003209. Epub 2013/01/26. PGENETICS-D-12-00212 [pii]. doi: 10.1371/journal.pgen.1003209 an advisor for Genapsys. No other competing interests exist. 23349633 PMC3547844

[35] Hildebrand EM, Biggins S. Regulation of Budding Yeast CENP-A levels Prevents Misincorporation at Promoter Nucleosomes and Transcriptional Defects. PLoS Genet. 2016;12(3):e1005930. Epub 20160316. doi: 10.1371/journal.pgen.1005930 . 26982580 PMC4794243

[36] Athwal RK, Walkiewicz MP, Baek S, Fu S, Bui M, Camps J, et al. CENP-A nucleosomes localize to transcription factor hotspots and subtelomeric sites in human cancer cells. Epigenetics Chromatin. 2015;8:2. Epub 20150113. doi: 10.1186/1756-8935-8-2 . 25788983 PMC4363203

[37] Earnshaw WC. Discovering centromere proteins: from cold white hands to the A, B, C of CENPs. Nat Rev Mol Cell Biol. 2015;16(7):443–9. Epub 2015/05/21. nrm4001 [pii] doi: 10.1038/nrm4001 . 25991376

[38] Stellfox ME, Bailey AO, Foltz DR. Putting CENP-A in its place. Cell Mol Life Sci. 2013;70(3):387–406. Epub 2012/06/26. doi: 10.1007/s00018-012-1048-8 . 22729156 PMC4084702

[39] Zeitlin SG. Centromeres: the wild west of the post-genomic age. Epigenetics. 2010;5(1):34–40. Epub 2010/01/23. 10629 [pii]. doi: 10.4161/epi.5.1.10629 . 20093854

[40] Liu X, Wang H, Zhao G. Centromere Protein A Goes Far Beyond the Centromere in Cancers. Mol Cancer Res. 2022;20(1):3–10. Epub 20210831. doi: 10.1158/1541-7786.MCR-21-0311 . 34465586

[41] Chuang C, Prasanth KR, Nagy PD. Coordinated Function of Cellular DEAD-Box Helicases in Suppression of Viral RNA Recombination and Maintenance of Viral Genome Integrity. PLoS Pathog. 2015;11(2):e1004680. Epub 2015/02/19. [pii]. doi: 10.1371/journal.ppat.1004680 . 25693185 PMC4333740

[42] Barajas D, Martin IF, Pogany J, Risco C, Nagy PD. Noncanonical Role for the Host Vps4 AAA+ ATPase ESCRT Protein in the Formation of Tomato Bushy Stunt Virus Replicase. PLoS Pathog. 2014;10(4):e1004087. Epub 2014/04/26. [pii]. doi: 10.1371/journal.ppat.1004087 . 24763736 PMC3999190

[43] Kovalev N, Pogany J, Nagy PD. A Co-Opted DEAD-Box RNA Helicase Enhances Tombusvirus Plus-Strand Synthesis. PLoS Pathog. 2012;8(2):e1002537. [pii]. doi: 10.1371/journal.ppat.1002537 . 22359508 PMC3280988

[44] Wang RY, Stork J, Pogany J, Nagy PD. A temperature sensitive mutant of heat shock protein 70 reveals an essential role during the early steps of tombusvirus replication. Virology. 2009;394(1):28–38. Epub 2009/09/15. S0042-6822(09)00481-4 [pii] doi: 10.1016/j.virol.2009.08.003 . 19748649 PMC2776036

[45] Wang RY, Stork J, Nagy PD. A key role for heat shock protein 70 in the localization and insertion of tombusvirus replication proteins to intracellular membranes. J Virol. 2009;83(7):3276–87. Epub 2009/01/21. JVI.02313-08 [pii] doi: 10.1128/JVI.02313-08 . 19153242 PMC2655559

[46] Pogany J, Stork J, Li Z, Nagy PD. In vitro assembly of the Tomato bushy stunt virus replicase requires the host Heat shock protein 70. Proc Natl Acad Sci U S A. 2008;105(50):19956–61. Epub 2008/12/09. 0810851105 [pii] doi: 10.1073/pnas.0810851105 . 19060219 PMC2604936

[47] Li Z, Vizeacoumar FJ, Bahr S, Li J, Warringer J, Vizeacoumar FS, et al. Systematic exploration of essential yeast gene function with temperature-sensitive mutants. Nat Biotechnol. 2011;29(4):361–7. Epub 2011/03/29. nbt.1832 [pii] doi: 10.1038/nbt.1832 . 21441928 PMC3286520

[48] Nagy PD, Pogany J. Yeast as a model host to dissect functions of viral and host factors in tombusvirus replication. Virology. 2006;344(1):211–20. Epub 2005/12/21. S0042-6822(05)00586-6 [pii] doi: 10.1016/j.virol.2005.09.017 . 16364751

[49] Panavas T, Nagy PD. Yeast as a model host to study replication and recombination of defective interfering RNA of Tomato bushy stunt virus. Virology. 2003;314(1):315–25. Epub 2003/10/01. S0042682203004367 [pii] doi: 10.1016/s0042-6822(03)00436-7 . 14517084

[50] Jaag HM, Nagy PD. Silencing of Nicotiana benthamiana Xrn4p exoribonuclease promotes tombusvirus RNA accumulation and recombination. Virology. 2009;386(2):344–52. Epub 2009/02/24. S0042-6822(09)00042-7 [pii] doi: 10.1016/j.virol.2009.01.015 . 19232421

[51] Panavas T, Hawkins CM, Panaviene Z, Nagy PD. The role of the p33:p33/p92 interaction domain in RNA replication and intracellular localization of p33 and p92 proteins of Cucumber necrosis tombusvirus. Virology. 2005;338(1):81–95. Epub 2005/06/07. S0042-6822(05)00234-5 [pii] doi: 10.1016/j.virol.2005.04.025 . 15936051

[52] Pogany J, Nagy PD. Authentic replication and recombination of Tomato bushy stunt virus RNA in a cell-free extract from yeast. J Virol. 2008;82(12):5967–80. Epub 2008/04/18. JVI.02737-07 [pii] doi: 10.1128/JVI.02737-07 . 18417594 PMC2395147

[53] Kovalev N, Pogany J, Nagy PD. Template role of double-stranded RNA in tombusvirus replication. J Virol. 2014;88(10):5638–51. Epub 2014/03/07. JVI.03842-13 [pii] doi: 10.1128/JVI.03842-13 . 24600009 PMC4019106

[54] Pathak KB, Pogany J, Xu K, White KA, Nagy PD. Defining the roles of cis-acting RNA elements in tombusvirus replicase assembly in vitro. J Virol. 2012;86(1):156–71. Epub 2011/10/21. JVI.00404-11 [pii] doi: 10.1128/JVI.00404-11 . 22013057 PMC3255868

[55] Pogany J, White KA, Nagy PD. Specific binding of tombusvirus replication protein p33 to an internal replication element in the viral RNA is essential for replication. J Virol. 2005;79(8):4859–69. Epub 2005/03/30. 79/8/4859 [pii] doi: 10.1128/JVI.79.8.4859-4869.2005 . 15795271 PMC1069559

[56] Pogany J, Fabian MR, White KA, Nagy PD. A replication silencer element in a plus-strand RNA virus. EMBO J. 2003;22(20):5602–11. Epub 2003/10/09. doi: 10.1093/emboj/cdg523 . 14532132 PMC213777

[57] Pathak KB, Pogany J, Nagy PD. Non-template functions of the viral RNA in plant RNA virus replication. Curr Opin Virol. 2011;1(5):332–8. Epub 2012/03/24. S1879-6257(11)00118-0 [pii] doi: 10.1016/j.coviro.2011.09.011 . 22440835

[58] Morey L, Barnes K, Chen Y, Fitzgerald-Hayes M, Baker RE. The histone fold domain of Cse4 is sufficient for CEN targeting and propagation of active centromeres in budding yeast. Eukaryot Cell. 2004;3(6):1533–43. Epub 2004/12/14. doi: 10.1128/EC.3.6.1533-1543.2004 . 15590827 PMC539035

[59] Lin W, Liu Y, Molho M, Zhang S, Wang L, Xie L, et al. Co-opting the fermentation pathway for tombusvirus replication: Compartmentalization of cellular metabolic pathways for rapid ATP generation. PLoS Pathog. 2019;15(10):e1008092. Epub 2019/10/28. [pii]. doi: 10.1371/journal.ppat.1008092 . 31648290 PMC6830812

[60] Prasanth KR, Chuang C, Nagy PD. Co-opting ATP-generating glycolytic enzyme PGK1 phosphoglycerate kinase facilitates the assembly of viral replicase complexes. PLoS Pathog. 2017;13(10):e1006689. Epub 2017/10/24. [pii]. doi: 10.1371/journal.ppat.1006689 . 29059239 PMC5695612

[61] Chuang C, Prasanth KR, Nagy PD. The Glycolytic Pyruvate Kinase Is Recruited Directly into the Viral Replicase Complex to Generate ATP for RNA Synthesis. Cell Host Microbe. 2017;22(5):639–52 e7. Epub 2017/11/07. S1931-3128(17)30439-0 [pii] doi: 10.1016/j.chom.2017.10.004 . 29107644

[62] Sasvari Z, Izotova L, Kinzy TG, Nagy PD. Synergistic roles of eukaryotic translation elongation factors 1Bgamma and 1A in stimulation of tombusvirus minus-strand synthesis. PLoS Pathog. 2011;7(12):e1002438. Epub 2011/12/24. [pii]. doi: 10.1371/journal.ppat.1002438 . 22194687 PMC3240602

[63] Li Z, Pogany J, Tupman S, Esposito AM, Kinzy TG, Nagy PD. Translation elongation factor 1A facilitates the assembly of the tombusvirus replicase and stimulates minus-strand synthesis. PLoS Pathog. 2010;6(11):e1001175. Epub 2010/11/17. doi: 10.1371/journal.ppat.1001175 . 21079685 PMC2973826

[64] Gasch P, Fundinger M, Müller JT, Lee T, Bailey-Serres J, Mustroph A. Redundant ERF-VII Transcription Factors Bind to an Evolutionarily Conserved cis-Motif to Regulate Hypoxia-Responsive Gene Expression in Arabidopsis. Plant Cell. 2016;28(1):160–80. Epub 2015/12/17. doi: 10.1105/tpc.15.00866 . 26668304 PMC4746684

[65] Hess N, Klode M, Anders M, Sauter M. The hypoxia responsive transcription factor genes ERF71/HRE2 and ERF73/HRE1 of Arabidopsis are differentially regulated by ethylene. Physiol Plant. 2011;143(1):41–9. Epub 2011/05/28. doi: 10.1111/j.1399-3054.2011.01486.x . 21615413

[66] Licausi F, van Dongen JT, Giuntoli B, Novi G, Santaniello A, Geigenberger P, et al. HRE1 and HRE2, two hypoxia-inducible ethylene response factors, affect anaerobic responses in Arabidopsis thaliana. Plant J. 2010;62(2):302–15. Epub 2010/02/02. doi: 10.1111/j.1365-313X.2010.04149.x . 20113439

[67] Nagy PD, Lin W. Taking over Cellular Energy-Metabolism for TBSV Replication: The High ATP Requirement of an RNA Virus within the Viral Replication Organelle. Viruses. 2020;12(1). Epub 2020/01/18. v12010056 [pii] doi: 10.3390/v12010056 . 31947719 PMC7019945

[68] Imamura H, Nhat KP, Togawa H, Saito K, Iino R, Kato-Yamada Y, et al. Visualization of ATP levels inside single living cells with fluorescence resonance energy transfer-based genetically encoded indicators. Proc Natl Acad Sci U S A. 2009;106(37):15651–6. Epub 2009/09/02. 0904764106 [pii] doi: 10.1073/pnas.0904764106 . 19720993 PMC2735558

[69] Kovalev N, de Castro Martin IF, Pogany J, Barajas D, Pathak K, Risco C, et al. Role of Viral RNA and Co-opted Cellular ESCRT-I and ESCRT-III Factors in Formation of Tombusvirus Spherules Harboring the Tombusvirus Replicase. J Virol. 2016;90(7):3611–26. Epub 2016/01/23. JVI.02775-15 [pii] doi: 10.1128/JVI.02775-15 . 26792735 PMC4794697

[70] de Castro IF, Fernandez JJ, Barajas D, Nagy PD, Risco C. Three dimensional imaging of the intracellular assembly of a functional viral RNA replicase complex. J Cell Sci. 2016. Epub 2016/03/31. jcs.181586 [pii] doi: 10.1242/jcs.181586 . 27026525

[71] Liu L, Li Y, Zhang S, Yu D, Zhu M. Hepatitis B virus X protein mutant upregulates CENP-A expression in hepatoma cells. Oncol Rep. 2012;27(1):168–73. Epub 2011/10/01. doi: 10.3892/or.2011.1478 . 21956590

[72] Himoto T, Tanaka N, Saito A, Muro Y, Sugiura K, Tani J, et al. Diversity of humoral responses to the centromere proteins among HCV-related chronic liver disease, PBC and AIH patients. Clin Res Hepatol Gastroenterol. 2015;39(2):222–9. Epub 2014/09/16. S2210-7401(14)00184-3 [pii] doi: 10.1016/j.clinre.2014.08.004 . 25220385

[73] Qin S, Liu Y, Tempel W, Eram MS, Bian C, Liu K, et al. Structural basis for histone mimicry and hijacking of host proteins by influenza virus protein NS1. Nat Commun. 2014;5:3952. Epub 2014/05/24. ncomms4952 [pii] doi: 10.1038/ncomms4952 . 24853335

[74] Mnaimneh S, Davierwala AP, Haynes J, Moffat J, Peng WT, Zhang W, et al. Exploration of essential gene functions via titratable promoter alleles. Cell. 2004;118(1):31–44. doi: 10.1016/j.cell.2004.06.013 . 15242642

[75] Dinesh-Kumar SP, Anandalakshmi R, Marathe R, Schiff M, Liu Y. Virus-induced gene silencing. Methods Mol Biol. 2003;236:287–94. Epub 2003/09/23. doi: 10.1385/1-59259-413-1:287 . 14501071

[76] Panaviene Z, Nagy PD. Mutations in the RNA-binding domains of tombusvirus replicase proteins affect RNA recombination in vivo. Virology. 2003;317(2):359–72. Epub 2003/12/31. S0042682203006627 [pii] doi: 10.1016/j.virol.2003.08.039 . 14698673

[77] Xu K, Nagy PD. Enrichment of Phosphatidylethanolamine in Viral Replication Compartments via Co-opting the Endosomal Rab5 Small GTPase by a Positive-Strand RNA Virus. PLoS Biol. 2016;14(10):e2000128. Epub 2016/10/21. doi: 10.1371/journal.pbio.2000128 . 27760128 PMC5070881

[78] Jonczyk M, Pathak KB, Sharma M, Nagy PD. Exploiting alternative subcellular location for replication: tombusvirus replication switches to the endoplasmic reticulum in the absence of peroxisomes. Virology. 2007;362(2):320–30. Epub 2007/02/13. doi: 10.1016/j.virol.2007.01.004 . 17292435

