# Supplementary material for: The centromeric histone CenH3 is recruited into the tombusvirus replication organelles
Source: PLoS Pathog. 2022 Jun 29;18(6):e1010653. doi: 10.1371/journal.ppat.1010653 (PMC9275711; doi:10.1371/journal.ppat.1010653)
Supplement: S1 Text — (DOCX) [file ppat.1010653.s001.docx]

## Supplementary materials and methods

***Yeast strains and expression plasmids.*** *Saccharomyces cerevisiae* strain BY4741 (*MAT*a, *his3Δ1, leu2Δ0, met15Δ0, ura3Δ0*) was obtained from Open Biosystems. The temperature-sensitive strain *cse4-1* was a generous gift from C. Boone (University of Toronto) (1). The following yeast expression plasmids have been previously described: HpGBK-CUP1-Hisp-33/ADH1-DI-72 (*HIS3* selection) (2), LpGAD-CUP1-His-p92 (*LEU* selection) (3). UpESC-GAL10-DI72/GAL1-His-p33 (*URA3* selection), TpGAD-CUP1-His-92 (*TRP1* selection) (4). HpGBK-CUP1-His-p33/GAL1-DI-72 (*HIS3* selection), HpGBK-CUP1-Flag-p33/GAL1-DI-72 (*HIS3* selection), LpGAD-CUP1-His-p92 (*LEU2* selection), LpGAD-CUP1-Flag-p92 (*LEU2* selection) (5). HpESC-GAL1-Hisp36/GAL10-DI-72 (*HIS3* selection) (6). LpESC-CUP1-Flag-CIRVp95 to be described elsewhere (*LEU2* selection, J. Pogany and P.D. Nagy). Overexpression plasmid pGAL-myc-CSE4 was donated by Dr. Sue Biggins (Fred Hutchinson Cancer Research Center) (7). Plasmid UpESC-His-AtCENH3, used for overexpression of *A. thaliana* CENH3 in yeast, was created by the PCR amplification from total *A. thaliana* cDNA with primers #6376/#6377 followed by insertion into UpESC vector after digestion of both the PCR and the vector with *EcoR*I and *Bgl*II restriction enzymes.

For expression of the Cse4 truncation mutants (see Fig. S2A) in yeast and *E. coli*, reverse primer #6809 and forward primers #6329 (for the full-length Cse4), #7080 (for cse4ΔN50), #7081 (for cse4ΔN80), #7083 (for cse4ΔN129) were used for the PCR-amplification of the Cse4 fragments missing various ΔN sequences, whereas forward primer #6329 and reverse primers #7112 (for cse4ΔC60) and #7113 (for cse4ΔC100) were used for the PCR-amplification of the ΔC fragments of Cse4. All PCR products were digested with *BamH*I and *Xho*I restriction enzymes and inserted into similarly digested pYES vector (for expression of His-tagged proteins in yeast) generating plasmids UpYES-His-CSE4, UpYES-His-cse4ΔN50, UpYES-His-cse4ΔN80, UpYES-His-cse4ΔN129, UpYES-His-cse4ΔC60 and UpYES-His-cse4ΔC100. The same *BamH*I/*Xho*I digested truncation fragments were also cloned into pGEX-His-RE and pMALc2X vectors (previously digested with *BamH*I/*Xho*I) for expression of GST or MBP fusion proteins in *E. coli*, generating plasmids pGEX-CSE4, pGEX-cse4ΔN50, pGEX-cse4ΔN80, pGEX-cse4ΔN129, pGEX-cse4ΔC60, pGEX-cse4ΔC100, pMAL-CSE4, pMAL-cse4ΔN50, pMAL-cse4ΔN80, pMAL-cse4ΔN129, pMAL-cse4ΔC60 and pGEX-cse4ΔC100. Similarly, AtCENH3 PCR product from total *A. thaliana* cDNA with primers #6331/#6609, was inserted into pGEX-his RE plasmid after digestion with *BamH*I and *Xho*I, generating plasmid pGEX-AtCENH3.

For expression of 6xHis-tagged Histone H3 in yeast, the primers #7116/#7117 were used to amplify Histone H3 sequence from *S. cerevisiae* total cDNA. The product was digested with *BamH*I and *Xho*I restriction enzymes, followed by cloning into equally digested pYES vector, generating plasmid UpYES-His-Histone H3.

The plasmid HpESC-GAL1-CFP-p33/GAL1-DI72 (*HIS3* selection) (8) was used for confocal laser microscopy as well as plasmid UpYES-GAL1-YFP-CSE4, which was created by PCR-amplification of the YFP sequence with primers #1291/#1295 using UpYES-YFP-p92 as template. The PCR product was then cloned into UpYES-His-CSE4 plasmid at the *BamH*I site.

***N. benthamiana plant expression plasmids*.** The plant expression plasmids pGD-T33-BFP, pGD-RFP-SKL, pGD-Cox4-RFP, pGD-p19 and pGD-C36-BFP have been described before (6, 9). VIGS plasmids were created as follows: The *Nicotiana tabacum* sequence for *CENH3* was used to do a blast in Benthgenome database (Queensland University of Technology) and obtain the predicted cDNA sequence. To generate the VIGS constructs, 5’ or 3’ fragments of the *NbCENH3* were PCR-amplified from total *N. benthamiana* cDNA using primer pairs #6380/6381 and #6382/6383. The PCR-fragments were inserted into the plasmid pTRV2 (10) to generate pTRV2-5’CENH3 and pTRV2-3’CENH3. For plasmids pGD-AtCENH3 and pGD-NbCENH3, PCR products were obtained with primers #6378/#6379 from *A. thaliana* or *N. benthamiana* cDNA followed by digestion of the products with *Xho*1 and *Sal*I restriction enzymes for insertion into pGD empty vector, generously donated by Dr. Michael Goodin (University of Kentucky) (11). Plasmids pGD-p33-ATeamYEMK and pGD-p36-ATeamYEMK have been described (12).

***Yeast transformation and cultivation.*** Yeast strains were co-transformed with different combinations of plasmids using the lithium acetate (LiAc)–single-stranded DNA (ssDNA)–polyethylene glycol (PEG) method (13), and transformants were selected by complementation of auxotrophic markers by plating them on selective SC medium.

***RNA analysis.*** Total RNA isolation and northern blot analysis were performed as described previously (3, 14) with a minor modification. Briefly, for extraction of total RNA, yeast cells were broken by shaking for 1 to 2 min at rt with equal volumes of RNA extraction buffer (50 mM NaOAc [pH 5.2], 10 mM EDTA, and 1% sodium dodecyl sulfate [SDS]) and water-saturated phenol and then incubated for 4 min at 65 °C. After a short incubation on ice and centrifugation at 4 °C for 10 min at 12,000 x *g*, the aqueous phase (~200 µl) was transferred to phenol-chloroform (~250 µl volume) mixed in 1:1 ratio, followed by vortexing, centrifugation (4 °C for 10 min at 12,000 x *g*) and ethanol precipitation of the aqueous phase. The obtained RNA samples were separated on a 1.5% agarose gel and transferred to a Hybond-XL membrane (Amersham) before hybridization with a ^32^P-labeled DI-72-specific or 18S ribosomal probe (14). Detection of (+)RNA was made via a ^32^P-labeled DI-72-RIII/IV probe prepared with in vitro T7-based transcription using PCR-amplified DNA obtained on pGBK-CUP1-6xHisp33/GAL1-DI-72 (5) template, with primers #22 and #1165. Viral RNA accumulation was normalized based on rRNA using the Image Quant software and a Typhoon scanner (General Electric).

Plant RNA isolation is almost the same as above with the difference that leaf discs are cut and frozen in liquid nitrogen, followed by a quick grinding (with a small blue pestle) before adding the RNA extraction buffer and phenol (15).

***Protein analysis by western Blot and immunodetection*.** For protein analysis, yeast strains were grown as for RNA extraction. Pelleted cells were resuspended in 200 µl of 0.1M NaOH and incubated at room temperature for 15 min while shaking in an Eppendorf shaker. NaOH was aspirated after a short centrifugation (1 min at 12,000 x *g*), and the pellets were re-suspended in 50 µl of 1X SDS/PAGE buffer with 5% β-mercaptoethanol. After other 15 min of shaking at room temperature, samples were incubated at 85 ºC for 15 min. After a 1 min centrifugation at 12,000 x *g* the supernatant was used for SDS/PAGE and western blot analysis as previously described (3, 16). To detect CNV, TBSV and CIRV viral proteins the primary antibody was anti-6xHis (Invitrogen), and the secondary antibody alkaline-phosphatase-conjugated anti-mouse IgG.

***Recombinant protein purification from E. coli*.** Recombinant proteins GST-Cse4, GST-AtCenH3, GST-cse4ΔN50, GST-cse4ΔN80, GST-cse4ΔN129, GST, MBP-p33C, MBP-p33, MBP-p92^pol^, MBP, MBP-Cse4, MBP-cse4ΔN50, MBP-cse4ΔN80, MBP-cse4ΔN129, MBP- cse4ΔC60 and MBP-cse4ΔC100 were expressed in *E. coli* and purified as described (17, 18). Briefly, *E. coli* strain BL21 (DE3) CodonPlus (Stratagene) cells were transformed with the above plasmids to express the recombinant proteins. The cells were then cultured at 37°C for 16 h overnight in 2 ml of MB medium with 100 μg/ml of ampicillin and 34 μg/ml of chloramphenicol. The culture was then diluted with fresh MB medium with antibiotics to adjust the concentration to OD_600_ ~0.2. After dilution, cultures were incubated at 37°C until reaching OD_600_ ~1. Subsequently, the cultures were incubated at 16°C for 8 h in the presence of isopropyl-β-D-thiogalactopyranoside (IPTG). The cells were then collected by centrifugation at 2,500 x *g* at 4°C for 5 min, followed by the resuspension with ice-cold column buffer (20mM HEPES [pH7.4], 25 mM NaCl, 1mM EDTA [pH 8.0]) containing 10 mM β-mercaptoethanol and 1 μg of RNase A for each 4 ml of cell suspension. Sonication was performed on ice to get the cell lysates, followed by centrifugation at 12,000 x *g* at 4°C for 15 min. The obtained supernatant was incubated with GST bind resin (EMD Millipore) for GST fusion proteins or amylose resin (NEB) for MBP fusion proteins at 4°C for 2 h, respectively. The resin was then washed with ice-cold column buffer four times. The recombinant protein was eluted with column buffer containing 10 mM glutathione and 1mM DTT in pH 7.5 for GST fusion proteins or 0.36% [W/V] maltose and 1mM DTT for MBP fusion proteins.

***Yeast cell free extract (CFE) based in vitro replication assay.*** This assay was prepared using BY4741 yeast strain as described previously (19, 20). Yeasts were grown at 23 ºC, reaching OD_600_ ~0.2, followed by heat treatment for 1 h at 37 ºC. The individual CFE preparations were then adjusted to contain comparable amounts of total proteins. The *in vitro* CFE reactions were prepared in 20 μl total volume containing 1 μl of adjusted CFE, 0.5 μg DI-72 (+)RNA transcripts, 0.5 μg affinity purified MBP-p33, 0.5 μg affinity-purified MBP-p92^pol^, 30 mM HEPES-KOH, pH 7.4, 150 mM potassium acetate, 5 mM magnesium acetate, 0.13 M sorbitol, 0.4 μl actinomycin-D (5 mg/ml), 2 μl of 150 mM creatine phosphate, 0.2 μl of 10 mg/ml creatine kinase, 0.2 μl of RNase inhibitor, 0.2 μl of 1 M dithiothreitol (DTT), 2 μl of 10 mM ATP, CTP, and GTP and 0.1 mM UTP, 0.1 μl of ^32^P-UTP and 0.1, 0.2 or 0.4 μg of affinity purified GST-AtCENH3 or GST as control. Reaction mixtures were incubated for 3 h at 25 ºC, followed by phenol/chloroform extraction and isopropanol/ammonium acetate (10:1) precipitation. ^32^P-UTP RNA products were analyzed in 5% acrylamide/8 M urea gels (19, 20).

***Gel mobility shift assay (EMSA) and RNA competition assay*.** Labeled RNAs for gel mobility shift experiments were prepared *in vitro* using T7 RNA polymerase. The labeled RNA probes were obtained using ^32^P-UTP in the T7 transcription reaction followed by removal of free nucleotides using micro-Bio-Spin columns (Bio-Rad). Template DNA was removed by DNase I, followed by purification of the RNA transcript with phenol-chloroform extraction and isopropanol precipitation. The pellet was washed with 70% ethanol to remove residual salts. The RNA transcripts were quantified by UV spectrophotometry with a Thermo Scientific NanoDrop. EMSA assays have been described previously (21). Briefly, the assay was performed with 0.1 pmol of 32P-labeled RNA probes along with different concentrations (0.1, 0.2 or 0.4 μM) of purified recombinant GST fusion proteins or GST in the presence of RNA binding buffer (10 mM HEPES [pH7.4], 50 mM NaCl, 1 mM DTT, 1 mM EDTA, 5% Glycerol, 2.5 mM MgCl_2_), 2 U of RNase inhibitor, as well as 0.1 μg of tRNA in a total of 10 μl reaction volume. After incubation of the reactions at 25ºC for 30 min the samples were analyzed by 5% nondenaturing PAGE performed at 200 V in Tris-borate-EDTA buffer for 1 h in a cold room.

Both labeled and unlabeled RNAs for the template competition experiments were prepared *in vitro* using T7 RNA polymerase. Two different amounts (2 and 4 pmol) of unlabeled RNAs (representing one of four regions of TBSV DI-72 RNA), together with 0.2 μM of either GST or GST-AtCenH3 were used for template competition in combination with the ^32^P-labeled (+) repRNA or (-) repRNA template (~0.1 pmol). Once again, the binding reaction was performed in the presence of RNA binding buffer (10 mM HEPES [pH7.4], 50 mM NaCl, 1 mM DTT, 1 mM EDTA, 5% Glycerol, 2.5 mM MgCl_2_), 2 U of RNase inhibitor, as well as 0.1 μg of tRNA. After incubation of the reactions at 25ºC for 30 min the samples were analyzed by 5% nondenaturing PAGE performed at 200 V in Tris-borate-EDTA buffer for 1 h in a cold room.

***dsRNA strand-separation assay*.** Preparation of dsRNA samples was performed as described (17). Briefly, unlabeled single-stranded DI-72 (+)repRNA was synthesized via T7 polymerase-based *in vitro* transcription. The ^32^P-labeled single-stranded DI72(-) was synthesized by T7-based *in vitro* transcription using ^32^P-labeled UTP. To prepare partial dsRNA duplexes, 2 pmol of ^32^P -labeled DI72(-) RNA was annealed to 6 pmol of unlabeled DI-72(+) RNA in STE buffer (10 mM TRIS [pH 8.0], 1 mM EDTA, and 100 mM NaCl) by slowly cooling down the samples (in a total volume of 20 μl) from 94°C to 25°C in 30 min.  Purified GST fusion proteins or GST as a negative control (2 μg) were added separately to the same amount of dsRNA duplex in the RNA binding buffer (10 mM HEPES [pH7.4], 50 mM NaCl, 1 mM DTT, 1 mM EDTA, 5% Glycerol, 2.5 mM MgCl_2_) along with 1mM ATP, followed by incubation at 25°C for 25 min. The reaction mixtures were then treated with Proteinase K (2 μg/per reaction) at 37°C for 20 min, followed by loading onto 5% nondenaturing polyacrylamide gel with 200V for 1 h in a cold room. The gels were dried, exposed and analyzed in a phosphoimager.

***Visualization and measurement of ATP levels in yeast*.** To analyze the ATP level in the TBSV replication compartment in yeast, BY4741 and cse4-1 cells were transformed with plasmids LpGAD-ADH-ATeam^YEMK^–p92^pol^, UpYC-GAL1-DI-72 and HpESC-GAL1-p33/GAL10-pex13-RFP (12). Transformed yeast cells were pre-grown in SC-ULH^-^ medium supplemented with 2% raffinose at 23 ºC for 12 h overnight and then washed with sterile water and resuspended in SC-ULH^-^ supplemented with 2% glucose for 1 h at 32 ºC. FRET images were obtained and analyzed as described above.

***Protein co-purification assays in yeast cells*.** For the co-purification of Cse4 with Flag-p33 replication protein from subcellular membranes, BY4741 yeast cells were co-transformed with plasmids HpGBK-CUP1-Flag-p33 (or HpGBK-CUP1-His-p33 as control), LpGAD-CUP1-Flag-p92 (or LpGAD-CUP1-His-p92 as a control), and UpGal-myc-CSE4. Cell growth and Flag-p33 purification from detergent-solubilized cellular membranes using anti-Flag M2 agarose was done as described previously (4)**.** Purified Flag-p33 was analyzed by western blot using anti-Flag antibody followed by anti-mouse antibody conjugated to alkaline phosphatase. Co-purified Myc-tagged proteins were analyzed with anti-Myc antibody followed by anti-mouse antibody conjugated to alkaline phosphatase. A similar approach was used for the co-purification of p33 with Flag-AtCenH3. Cells were transformed with plasmids HpGBK-CUP1-His-p33, LpGAD-CUP1-His-p92 and UpESC-GAL1-Flag-AtCENH3 (or UpESC-GAL1-His-AtCENH3 as a control). Cell growth and Flag-p33 purification using anti-Flag M2 agarose was done as described previously (4) but Flag-AtCenH3 was purified from the soluble fraction instead of the cellular membranes.

***Cse4 pull-down assay.*** This assay was performed as described previously (22). Briefly, *E. coli* expressing GST-tagged p33C or GST were resuspended in ice-cold column buffer (10 mM Tris-HCl [pH 7.4], 1 mM EDTA, 25 mM NaCl, 10 mM β-mercaptoethanol) and lysed by sonication. The cleared lysate was passed through a column containing glutathione resin to capture the GST-tagged viral proteins or GST (negative control). The columns were washed three times with cold column buffer prior to the addition of recombinant MBP fusion proteins purified from *E. coli* (see above). The same amount of MBP fusion proteins were loaded onto columns with captured GST-p33C or GST, followed by incubation at 4 ºC for 2 h. After washing the columns 5 times with chilled column buffer, the bound proteins were eluted with 50 ml SDS-PAGE sample buffer from the columns and analyzed by Western blotting using an anti-MBP antibody.

**References**

1. Li Z*, et al.* (2011) Systematic exploration of essential yeast gene function with temperature-sensitive mutants. *Nat Biotechnol* 29(4):361-367.

2. Mendu V, Chiu M, Barajas D, Li Z, & Nagy PD (2010) Cpr1 cyclophilin and Ess1 parvulin prolyl isomerases interact with the tombusvirus replication protein and inhibit viral replication in yeast model host. *Virology* 406(2):342-351.

3. Panaviene Z, Panavas T, Serva S, & Nagy PD (2004) Purification of the cucumber necrosis virus replicase from yeast cells: role of coexpressed viral RNA in stimulation of replicase activity. *Journal of virology* 78(15):8254-8263 .

4. Barajas D*, et al.* (2014) Co-opted oxysterol-binding ORP and VAP proteins channel sterols to RNA virus replication sites via membrane contact sites. *PLoS pathogens* 10(10):e1004388 .

5. Barajas D, Jiang Y, & Nagy PD (2009) A unique role for the host ESCRT proteins in replication of Tomato bushy stunt virus. *PLoS Pathog* 5(12):e1000705.

6. Xu K, Huang TS, & Nagy PD (2012) Authentic in vitro replication of two tombusviruses in isolated mitochondrial and endoplasmic reticulum membranes. *J Virol* 86(23):12779-12794.

7. Ranjitkar P*, et al.* (2010) An E3 ubiquitin ligase prevents ectopic localization of the centromeric histone H3 variant via the centromere targeting domain. *Mol Cell* 40(3):455-464.

8. Wang RY, Stork J, & Nagy PD (2009) A key role for heat shock protein 70 in the localization and insertion of tombusvirus replication proteins to intracellular membranes. *J Virol* 83(7):3276-3287.

9. Xu K & Nagy PD (2016) Enrichment of Phosphatidylethanolamine in Viral Replication Compartments via Co-opting the Endosomal Rab5 Small GTPase by a Positive-Strand RNA Virus. *PLoS Biol* 14(10):e2000128.

10. Dinesh-Kumar SP, Anandalakshmi R, Marathe R, Schiff M, & Liu Y (2003) Virus-induced gene silencing. *Methods Mol Biol* 236:287-294.

11. Goodin MM, Dietzgen RG, Schichnes D, Ruzin S, & Jackson AO (2002) pGD vectors: versatile tools for the expression of green and red fluorescent protein fusions in agroinfiltrated plant leaves. *Plant J* 31(3):375-383.

12. Chuang C, Prasanth KR, & Nagy PD (2017) The Glycolytic Pyruvate Kinase Is Recruited Directly into the Viral Replicase Complex to Generate ATP for RNA Synthesis. *Cell Host Microbe* 22(5):639-652 e637 .

13. Gietz RD & Woods RA (2002) Transformation of yeast by lithium acetate/single-stranded carrier DNA/polyethylene glycol method. *Methods Enzymol* 350:87-96.

14. Panavas T & Nagy PD (2003) Yeast as a model host to study replication and recombination of defective interfering RNA of Tomato bushy stunt virus. *Virology* 314(1):315-325 .

15. Jaag HM & Nagy PD (2009) Silencing of Nicotiana benthamiana Xrn4p exoribonuclease promotes tombusvirus RNA accumulation and recombination. *Virology* 386(2):344-352 .

16. Nawaz-ul-Rehman MS, Reddisiva Prasanth K, Baker J, & Nagy PD (2013) Yeast screens for host factors in positive-strand RNA virus replication based on a library of temperature-sensitive mutants. *Methods (San Diego, Calif.)* 59(2):207-216 .

17. Rajendran KS & Nagy PD (2003) Characterization of the RNA-binding domains in the replicase proteins of tomato bushy stunt virus. *J Virol* 77(17):9244-9258.

18. Kovalev N, Pogany J, & Nagy PD (2012) A Co-Opted DEAD-Box RNA helicase enhances tombusvirus plus-strand synthesis. *PLoS Pathog* 8(2):e1002537.

19. Pogany J & Nagy PD (2008) Authentic replication and recombination of Tomato bushy stunt virus RNA in a cell-free extract from yeast. *J Virol* 82(12):5967-5980.

20. Pogany J, Stork J, Li Z, & Nagy PD (2008) In vitro assembly of the Tomato bushy stunt virus replicase requires the host Heat shock protein 70. *Proc Natl Acad Sci U S A* 105(50):19956-19961.

21. Pogany J, White KA, & Nagy PD (2005) Specific binding of tombusvirus replication protein p33 to an internal replication element in the viral RNA is essential for replication. *J Virol* 79(8):4859-4869.

22. Barajas D, Li Z, & Nagy PD (2009) The Nedd4-type Rsp5p ubiquitin ligase inhibits tombusvirus replication by regulating degradation of the p92 replication protein and decreasing the activity of the tombusvirus replicase. *J Virol* 83(22):11751-11764.

**Table A: The sequences of primers used in this study**

| **Primer #** | **Sequence** |
| --- | --- |
| 22 | GTAATACGACTCACTATAGGGCTGCATTTCTGCAATGTTCC |
| 1165 | AGCGAGTAAGACAGACTCTTCA |
| 1291 | CGGCGGATCCGTGAGCAAGGGCGAGGAGCTGTTCA |
| 1295 | CGGCGGATCCCTTGTACAGCTCGTCCATGCCGA |
| 2030 | CGCGGGATCCATGTCAAAAGCTGTCGGTATTG |
| 2534 | GTAATACGACTCACTATAGGGAGCCACCAACAAGA |
| 2535 | TGTATGGAACCAGTTGAAAAGTGTTTGAGGG |
| 2859 | TAATACGACTCACTATAGGAACCAAATCATTCATGTTGCTCTC |
| 2860 | TAGTGTATGTGATATCCCACCAA |
| 4308 | CCAGACTAGTATGGCTGAACTGAGCGAACAAG |
| 5604 | GTAATACGACTCACTATAGGACCGTTACCCAAGGTGTGG |
| 5621 | CGCCGGATCCATGTCTGAAATTACTTTGGGTAAATA |
| 5992 | CGCCGGATCCATGTCTAGATTAGAAAGATTGA |
| 6275 | CGGGATCCCGATGTCTTTATCTTCAAAGTTG |
| 6329 | CGCCGGATCCATGTCAAGTAAACAACAATGGGTTAG |
| 6331 | CGCCCCATGGAATAAACTGTCCCCTGATTCTTCT |
| 6367 | TAATACGACTCACTATAGGGCTTACCCAACAATGATTGCA |
| 6376 | CGCCGAATTCATGCATCATCATCATCATCATGCGAGAACCAAGCATC |
| 6377 | CGCCAGATCTTCACCATGGTCTGCCTTTTC |
| 6378 | CGCCCTCGAGATGCATCATCATCATCATCATGCGAGAACCAAGCATC |
| 6379 | CGCCGTCGACTCACCATGGTCTGCCTTTTC |
| 6380 | CAACGGATCCATGGCGAGAACCAAACACCT |
| 6381 | CAACCTCGAGACAAGTCTGATGAAAGGAGCAGC |
| 6382 | CAACGGATCCTTTGCACCAGAGGTAACTCGC |
| 6383 | CAACCTCGAGTCACCAAGGTCGTGCTTTTC |
| 6609 | CGCCCTCGAGTCACCATGGTCTGCCTTTTC |
| 6809 | CGCCCTCGAGCTAAATAAACTGTCCCCTGATTCTTC |
| 8174 | GTGCTGAAATCGTGGAATCTG |
| 8175 | TCCATTCCCAATAGTCACACG |
| 8176 | AAGTGTGATCCTCTGTGCAG |
| 8177 | CAGATCCACAATTACTCCAGGG |
| 8178 | CTGGGAAGTTATCTGTGACGAG |
| 8179 | AACAGCCCTAGGAACATAACG |
| 8217 | ACGAGAAGGAATACAAGCCAG |
| 8218 | CAGTAAGGGAGTGGACAGTAG |
| 8219 | GGTATCTCTATTGCTTCCCACTG |
| 8220 | AATATCCAATCCCATCCAGCC |
| 8221 | TTGCCTTACCAGTTGTCTCG |
| 8222 | TCCCCTGTAAAGACCTGAATTG |
| 7080 | CGCCGGATCCCTGTTTCCAAGAAGAGAGGAAAG |
| 7081 | CGCCGGATCCCTAGAAATCGAGACAGAAAATGAAG |
| 7083 | CGCCGGATCCAAGAAATATACTCCTAGTGAATTAGCTCTG |
| 7112 | CGCCCTCGAGAAACTCGTCTGTAACTTCTTTCACTAG |
| 7113 | CGCCCTCGAGTTCGACGCGCTTTAAGCTC |
| 7116 | CGCCGGATCCATGGCCAGAACAAAGCAAAC |
| 7117 | CGCCCTCGAGCTATGATCTTTCACCTCTTAATCTTCTAG |
| 7123 | CGCCGGATCCATGGCTGTCTCTAAAGTTTACG |
| 7136 | TAATACGACTCACTATAGGGCAACATCGTTGGTGGTGGTAC |
| 7137 | TAATACGACTCACTATAGGGAGGACTTAGACAAGTCAGCCAAATG |
| 7138 | TAATACGACTCACTATAGGGAGGTTTCACCCTTAAATTCAACTTG |
| 7140 | TAATACGACTCACTATAGGGCATCGATCCATCTACCACCA |
| 7141 | AGGATGGTCAAACCAGAGAACAC |
| 7142 | TAATACGACTCACTATAGGGATGGAGCGTTGGTGGTAGC |
| 7295 | GTGATCTCATTGGAATAGCAGAAACA |
| 7296 | TAATACGACTCACTATAGGGAGCAGCAACATCTGTAGCTGTC |
| 7297 | TTGAGACCACCAAGTACTACTGC |
| 7298 | TAATACGACTCACTATAGGGTGGAAGCAACAAACCCAC |
