# Supplementary figures and images for: The centromeric histone CenH3 is recruited into the tombusvirus replication organelles
Source: PLoS Pathog. 2022 Jun 29;18(6):e1010653. doi: 10.1371/journal.ppat.1010653 (PMC9275711; doi:10.1371/journal.ppat.1010653)

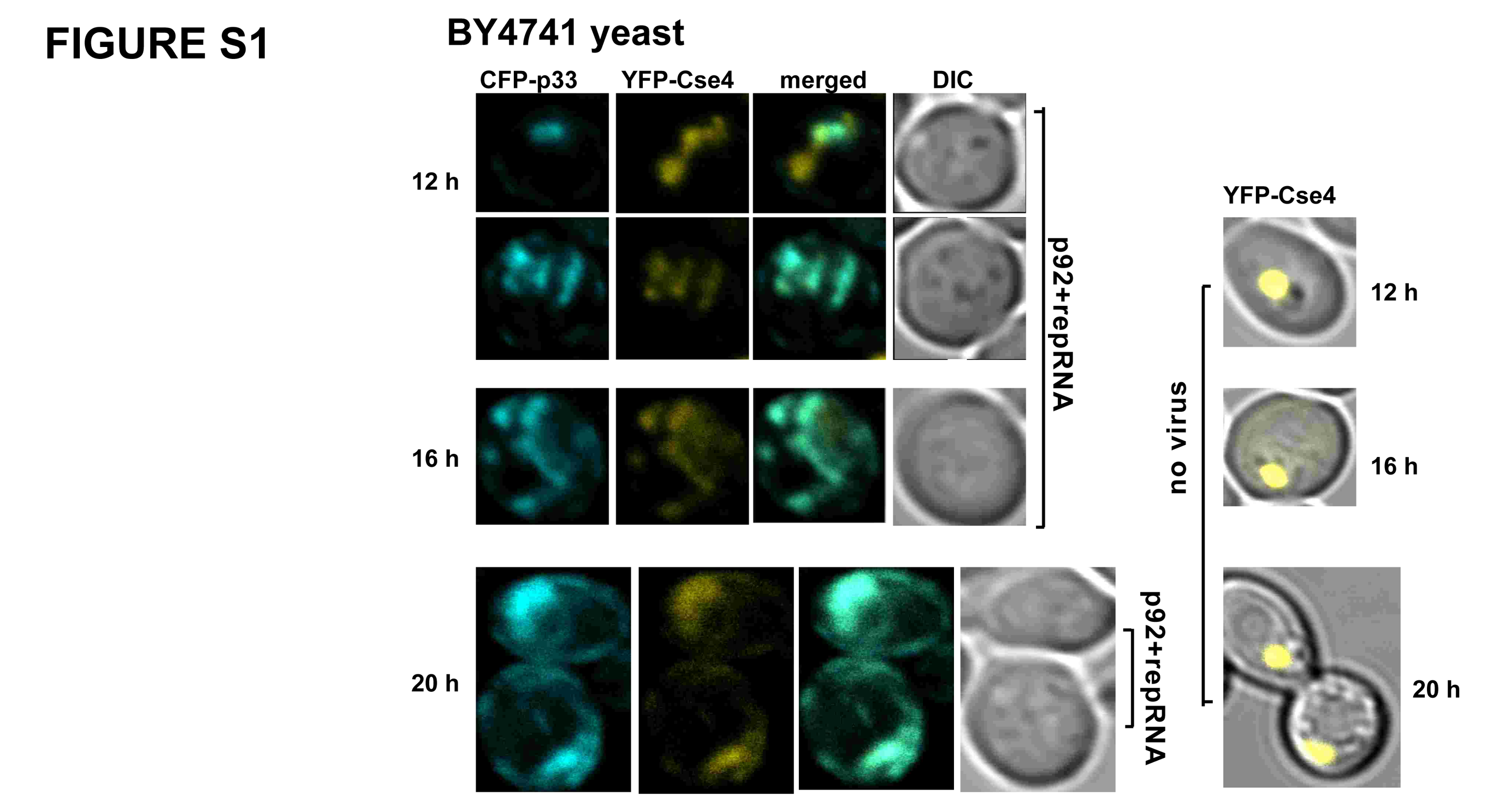

Supplement: S1 Fig — Confocal laser microscopy analyses in WT yeast cells co-expressing YFP-Cse4 together with CFP-p33, p92pol replication proteins and the (+)repRNA show partial co-localization of YFP-Cse4 with CFP-p33 at 12 h, 16 h and 24 h after induction of protein expression. Images on the right show the nuclear distribution of YFP-Cse4 in the absence of viral components in WT yeast cells at the same time points. (TIF) [file ppat.1010653.s002.tif]

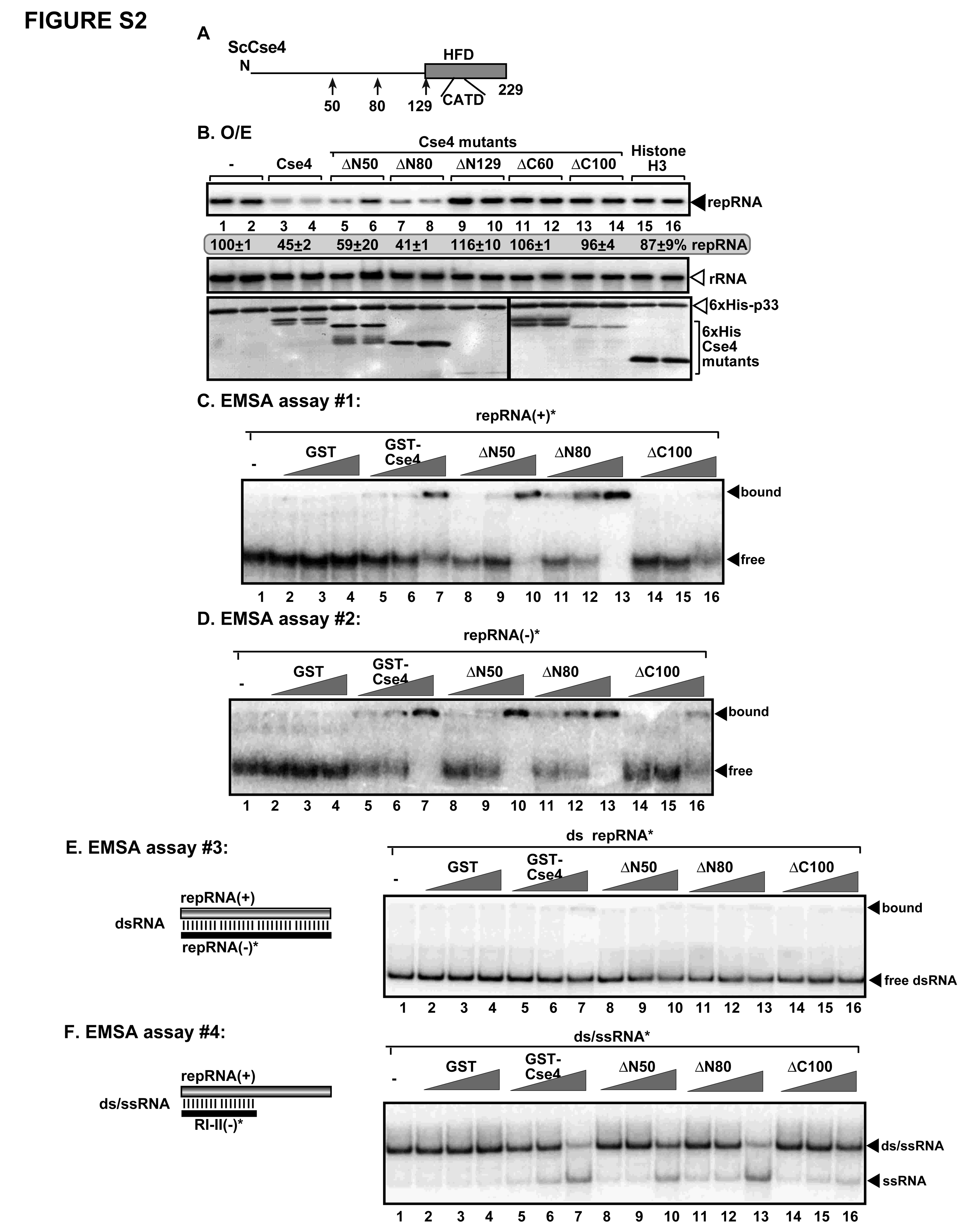

Supplement: S2 Fig — (A) Schematic diagram showing the endpoints of the Cse4 deletion mutants used in this study. The proteins were named by the number of the last amino acid deleted. (B) Top: northern blot analysis shows a reduction in the accumulation of repRNA in cells expressing full length Cse4 (lanes 3–4) and the N-terminal deletion mutants ΔN50 (lanes 5–6) and ΔN80 (lanes 7–8) compared to the control samples (lanes 1–2). Expression of ΔN129, ΔC60, ΔC100 and Histone H3 did not affect TBSV repRNA accumulation (lanes 9–16). Middle: Northern blot with 18S ribosomal RNA specific probe was used as a loading control. Bottom: Western blot analyses of the level of His6-p33 replication protein and His6-Cse4 mutants with anti-His antibody. Note that levels of ΔN129 are very low, suggesting that the N-terminal region of Cse4 has a role in protein stability. (C, D) RNA gel mobility shift analysis shows that GST-Cse4, GST-ΔN50 and GST-ΔN80 efficiently bind to 32P-labeled (+)repRNA (C) or (-)repRNA (D) in vitro, whereas GST-ΔC100 show defective binding capability to both repRNAs. Purified GST-Cse4, GST-ΔN50, GST-ΔN80, GST-ΔC100 and GST were added in increasing concentrations (0.1, 0.2 or 0.4 μM) to the assays. The 32P-labeled ssRNA—protein complexes were visualized on nondenaturing 5% polyacrylamide gels. (E, F) RNA-strand separation assays. Left: Schematic representation of the RNA/RNA duplexes used in the assays. See details in Fig 3F and 3G. Right: Increasing amounts (0.1, 0.2 or 0.4 μM) of purified recombinant GST-Cse4, GST-ΔN50, GST-ΔN80, GST-ΔC100 and GST (as a control), were added to the assays. The 32P-labeled RNA products after the in vitro RNA-strand separation assay were analyzed on nondenaturing 5% polyacrylamide gels. Full length Cse4 and all mutants were unable to unwind a fully duplexed dsRNA (E), whereas WT Cse4 and the N-terminal deletion mutants unwound the partial dsRNA (F). Three independent repeats were performed for each experiment. (TIF) [file ppat.1010653.s003.tif]

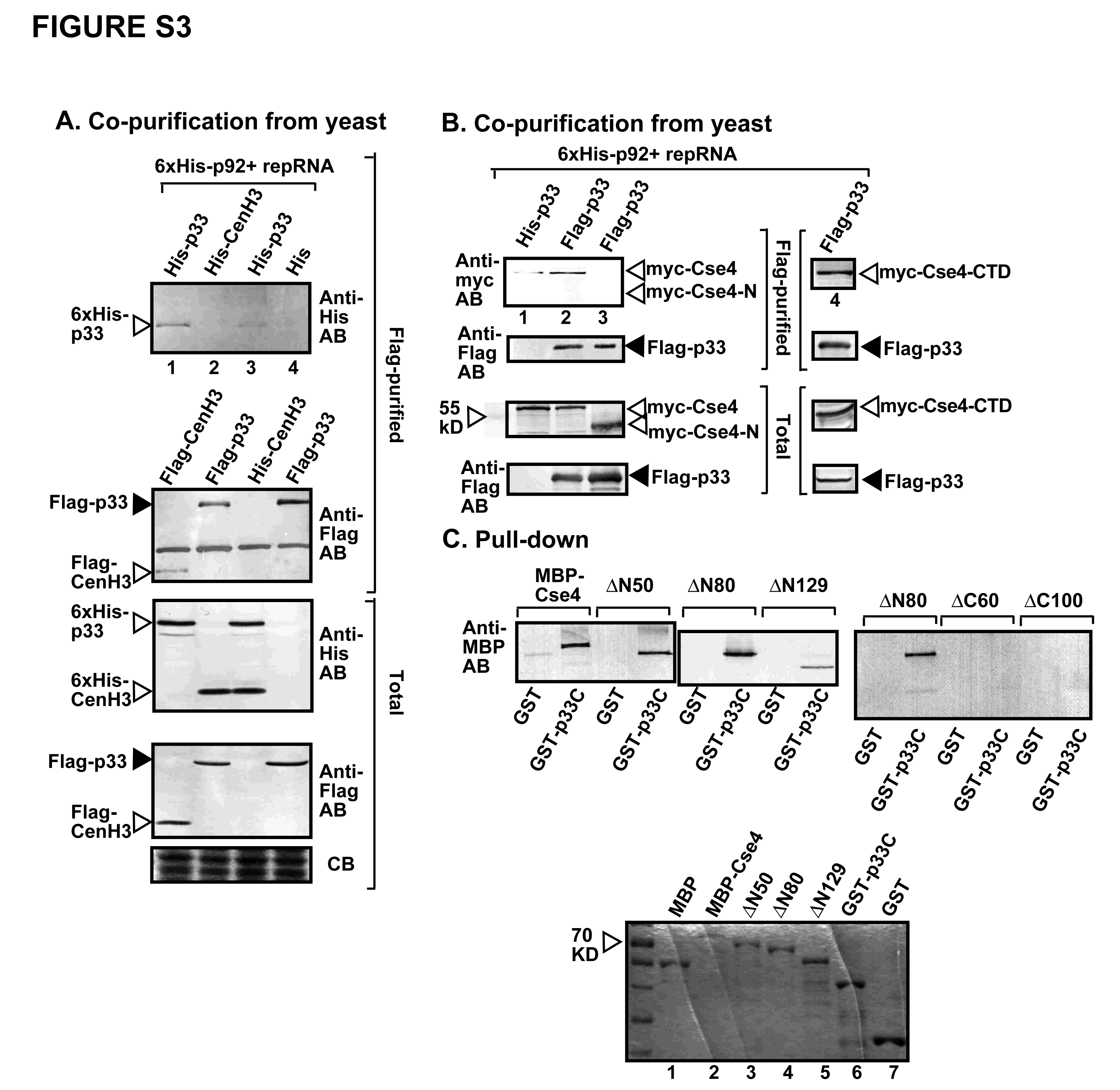

Supplement: S3 Fig — (A) Co-purification of viral p33 replication protein with plant CenH3. First panel: western blot analysis of co-purified His6-p33 with Flag-affinity purified A. thaliana Flag-CenH3 from membrane fraction of WT yeast. His6-p33 was detected with anti-His antibody. The negative control was His6-tagged AtCenH3 which was not co-purified from yeast extracts when using a Flag-affinity column (lane 2). Second panel: Western blot of purified Flag-AtCenH3 and Flag-p33 detected with anti-Flag antibody. Bottom panels: Western blot of His6- or Flag-tagged proteins in total yeast extracts. (B) Co-purification of yeast Cse4p with the viral replicase complex. First panels: western blot analysis of co-purified Myc-tagged Cse4 (lane 2) and Cse4 C-terminal domain (Cse4-CTD, lane 4) with Flag-affinity purified Flag-p33 from WT yeast membrane fraction. Myc-Cse4 and Myc-Cse4-CTD were detected with anti-Myc antibody. The negative control was His6-tagged p33 (lane 1). Second panels: Western blots of purified Flag-p33 detected with anti-Flag antibody. Bottom panels: Western blot of Myc-Cse4, Myc-Cse4-CTD, Myc-Cse4 N terminal domain (Myc-Cse4-N) and Flag-tagged p33 in the total yeast extracts. Note that after affinity-purification, Myc-Cse4-N was not co-purified with p33 (lane 3). (C) Pull-down assay including TBSV GST-p33 replication protein and the MBP-tagged Cse4 or Cse4 deletion mutants. The C-terminal region of TBSV p33 replication protein was used instead of the full-length protein, which includes the non-soluble N-terminal region with the trans-membrane domains. Top: Western blot analysis of the captured GST-p33C with MBP purified WT GST-Cse4 or ΔN50, ΔN80, ΔN129 Cse4 deletion mutants. GST was used as a control. Note that similar to Myc-Cse4-N, the C-terminal deletion mutants ΔC60 and ΔC100 were not pulled-down with GST-p33C suggesting that the HFD of Cse4 is also important for the interaction between p33 replication protein and Cse4p. Bottom: Coomassie-blue stained SDS-PAGE of the pu [file ppat.1010653.s004.tif]

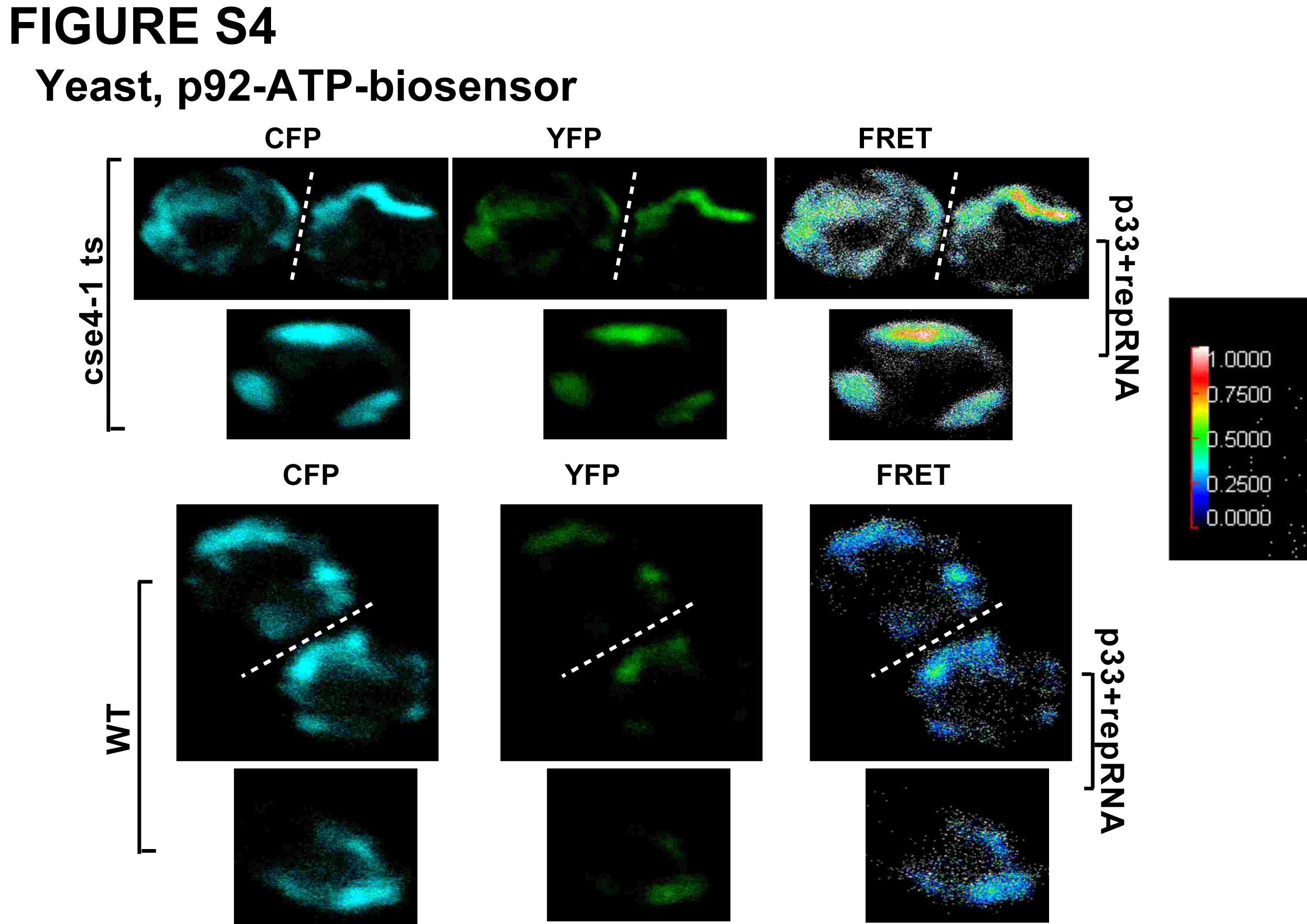

Supplement: S4 Fig — Comparison of the ATP level within the tombusvirus replication compartment in WT and cse4-1 yeasts grown at 23°C using ATeamYEMK–p92pol. See further details in Fig 5. Increased generation of ATP within VROs was observed in cse4-1 temperature sensitive strain compared to control WT yeast grown under the same conditions. White dashed lines separate two yeast cells. Images are representative of two independent experiments. (TIF) [file ppat.1010653.s005.tif]
